# Virtual screening and molecular dynamics of anti-Alzheimer compounds from *Cardiospermum halicacabum* via GC-MS

**DOI:** 10.3389/fchem.2025.1586728

**Published:** 2025-04-04

**Authors:** Selvan Kaviyarasu, Nallamuthu Padmanaban, Sulekha Khute, Gokhan Zengin, Paranthaman Subash

**Affiliations:** ^1^ Department of Pharmacognosy, Sri Shanmugha College of Pharmacy, Sankari, Tamil Nadu, India; ^2^ Department of Biology, Science Faculty, Selcuk University, Konya, Türkiye

**Keywords:** Alzheimer’s disease, *Cardiospermum halicacabum*, GC-MS, phytoconstituents, molecular dynamics, HOMO-LUMO

## Abstract

**Background:**

Ayurveda is an ancient Indian medicinal system that uses medicinal plants for their neuroprotective effects. Ayurveda claims that the (*C. halicacabum*) leaves possess significant neuroprotective properties. Alzheimer’s is characterized by the accumulation of amyloid-β, acetylcholinesterase, and tau tangles that interfere with neural transmission and impair cognitive abilities.

**Objectives:**

This study aimed to identify novel potential anti-Alzheimer phytoconstituents of *C. halicacabum* leaves using *in silico* methods.

**Methods:**

This study utilized the Box–Behnken design within the response surface methodology (RSM) to optimize and combine the effects of process variables, namely powder weight, solvent volume, and extraction time, on the microwave-assisted extraction (MAE) *of C. halicacabum* leaves. The optimization process revealed that these variables, along with microwave usage, significantly influenced the extraction yield. The ethanolic extract was examined using gas chromatography-mass spectrometry (GC–MS) analysis, and the identified phytoconstituents were further analyzed through computer-based simulations, including docking, absorption, distribution, metabolism, excretion, and toxicity (ADMET) studies, assessment of drug-likeness, molecular dynamics, LigPlot analysis, and density functional theory (DFT) analysis.

**Results:**

Gas chromatography-mass spectrometry (GC-MS) analysis identified 40 phytoconstituents and 37 were successfully characterized. Molecular docking and dynamics simulations revealed two lead compounds, acetic acid (dodecahydro-7-hydroxy-1,4b,8,8-tetramethyl-10-oxo-2(1H)-phenanthrenylidene)-,2-(dimethylamino)ethyl ester, [1R-(1. alpha)], and 1-(2-hydroxyethoxy)-2-methyldodecane, which exhibited superior stability in the docked complex compared to galantamine.

**Conclusion:**

Based on computational predictions and observed pharmacological properties, these findings suggest that phytoconstituents may have therapeutic effects against selected AD targets.

## 1 Introduction

Alzheimer’s disease (AD) is a neurodegenerative disorder characterized by the accumulation of amyloid beta peptide (Aβ), which leads to the formation of amyloid plaques that disrupt neuronal communication and cause cognitive decline. Hyperphosphorylated tau proteins aggregate into neurofibrillary tangles, further impairing neuronal function and leading to cell death. Acetylcholinesterase (AChE), which is essential for memory and learning, is disrupted in AD, exacerbating cognitive deficits in patients. Although there is no cure, early detection and treatment can improve the quality of life. Early detection and treatment can significantly improve the quality of life of individuals with AD (Reddy et al., 2025; Azargoonjahromi, 2024; Sarkar et al., 2024). Currently available therapies, such as NMDA receptor antagonists and acetylcholinesterase inhibitors, primarily treat neurodegenerative conditions, such as Alzheimer’s disease, rather than treating the disease itself (Shukla et al., 2024; Pardo-Moreno et al., 2022; Mathew et al., 2023). Recently, heteroaryl–acrylonitrile compounds have been identified as a new class of AChEI responsible for the breakdown of the neurotransmitter acetylcholine (Sun et al., 2024). Despite these drugs improving cognitive performance in AD, the development of the disease remains unaddressed, and further research is focused on targeted therapeutics for neurodegenerative diseases involving the Aβ, Tau, and cholinergic systems (Pardo-Moreno et al., 2022; Ornish et al., 2024; Razzaghi et al., 2024). These drug candidates can help delay changes that lead to clinical symptoms; however, they cannot prevent disease progression (Sun et al., 2022). The global incidence of AD is increasing, necessitating urgent efforts to identify effective treatment and prevention methods by 2025.

This public health challenge is not only emotional for families but also economic for the healthcare systems. The focus on disease-modifying therapies (DMTs) has shifted from managing symptoms to altering the disease progression and targeting the mechanisms underlying AD, such as Aβ plaques, acetylcholinesterase, and tau tangle accumulation. Researchers aim to delay disease progression and improve the wellbeing of patients using DMTs (Pedroza et al., 2022). The development of effective drugs for AD is formidable and is marked by intricate research and stringent testing. This process begins with understanding the complex pathology of the disease, which guides the identification of potential therapeutic targets. Recent advancements have led to the development of drugs such as lecanemab and donanemab, which focus on amyloid proteins that are believed to play a critical role in Alzheimer’s progression (Terao and Kodama, 2024). However, these drugs address only one aspect of the disease, highlighting the need for broader therapeutic approach. Promising candidates, such as UB-311 and HMTM, are still undergoing rigorous evaluation in clinical trials, a phase that spans several years (Czech and Schulz, 2024). These trials are crucial for assessing drug safety profiles, efficacy, and potential side effects over time. Certain medications, including cholinesterase inhibitors and NMDA receptor antagonists, can offer symptomatic relief, boost cognitive abilities, and possibly increase the quality of life; however, these treatments do not stop or reverse the progression of AD. Identification of effective treatments for the underlying conditions of this complex neurodegenerative disorder is essential (Alhazmi and Albratty, 2022). This restriction may be more successfully addressed by herbal medicine, which use a variety of secondary metabolites that target many proteins or signalling pathways and deliver synergistic benefits with few side effects (Salvoza et al., 2022).

**FIGURE 1:**
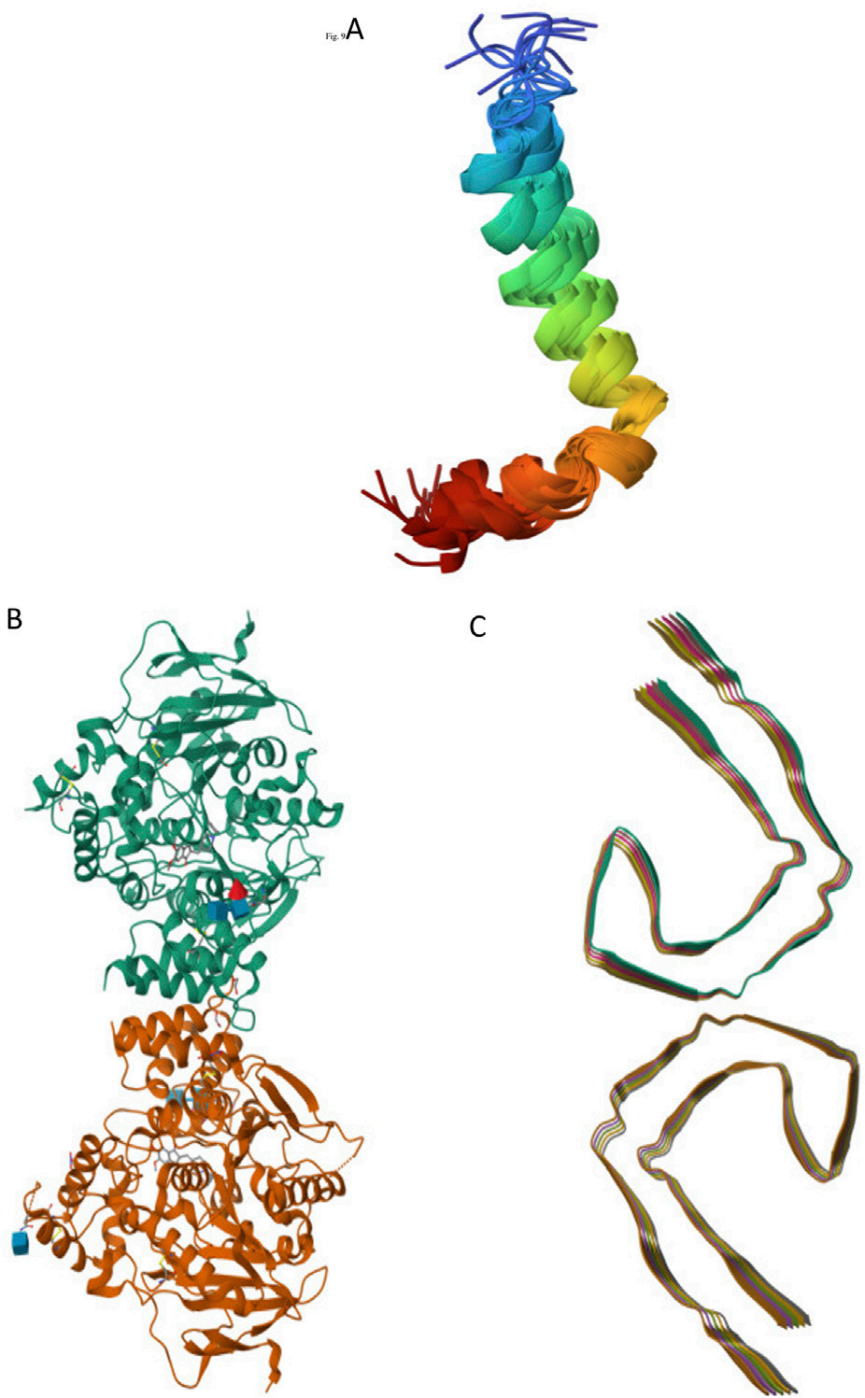
Selected Alzheimer’s targets: 1IYT-amyloid beta-peptide **(A)**, 4EY7-acetylcholinesterase **(B)**, and 5O3L-tau filaments **(C)**.

Recent studies have focused on the selection of targets associated with neurodegenerative diseases using advanced crystallographic techniques. The crystal structure of amyloid-β peptide (1–42), denoted by the identifier 1IYT (Figure 1A), provides crucial insights into the role of this peptide in AD, revealing its propensity to form plaques in the brain (Zou et al., 2024). Similarly, the crystal structure identified as 4EY7 (Figure 1B) corresponds to human AChE, an enzyme pivotal in nerve signal transmission that is often targeted by drugs aimed at alleviating AD symptoms by preventing the breakdown of neurotransmitters. Additionally, tau protein, with its structure labeled 5O3L (Figure 1C), is another critical component in neurodegenerative research, as its abnormal aggregation leads to the formation of neurofibrillary tangles, a hallmark of AD and other tauopathies (Hasan et al., 2023; Segura et al., 2024). Structural studies play a crucial role in elucidating the complexities of neurodegenerative diseases, such as AD and Parkinson’s disease. By examining the molecular and cellular architecture of the affected brain regions, researchers can identify the specific abnormalities and mechanisms that lead to neuronal damage and loss. This understanding is essential for the identifying potential therapeutic targets. Structural studies often employ advanced imaging techniques, such as magnetic resonance imaging (MRI) and cryo-electron microscopy to visualize brain structures in detail. These insights will enable the development of novel treatments that can slow or even halt disease progression, ultimately improving the quality of life of patients.

Natural products have emerged as promising alternatives owing to their ability to interact with multiple molecular targets and fewer side effects (Chaachouay and Zidane, 2024). *Cardiospermum halicacabum* L., belonging to the family Sapindaceae (referred to as the “balloon vine”), is a tropical and subtropical plant used to treat rheumatism, lumbago, neurological disorders, orchitis and dropsy. The leaves, aerial parts, and seeds of this plant are used to treat neurological diseases (Elangovan et al., 2022; Menichini et al., 2014). According to Ayurveda, the traditional medicinal plant *C. halicacabum* contains bioactive compounds with potential neuroprotective properties (Topiya and Pandya, 2024), and its leaves are primarily used for their neuroprotective properties. The Box–Behnken response surface methodology was used to understand the interaction between the different factors. In this study, we focused on the extraction of beneficial elements from *C. halicacabum* leaves using MAE. The ideal conditions for optimum extraction yields can be determined by combining solvent volume, powder weight, and extraction time. Researchers can optimize their extraction procedures and produce useful chemicals for various applications by varying factors such as powder weight, solvent ratio, and extraction period. This technique paves the way for further research on the use of compounds recovered from *C. halicacabum* leaves by improving the overall extraction yield and time. Moreover, esterification cannot be used to extract oil-containing hydrolyzable components for phytochemical extraction (Sonntag, 1982).

Microwave extraction is an innovative and efficient method for separating phytoconstituents from solid matrices, such as plant materials. This technique, known as MAE, exploits the ability of microwaves to penetrate the matrix and rapidly heat the moisture. This results in the cellular breakdown of plant materials, allowing for the rapid release of bioactive compounds (Ferrara et al., 2023; Bhadange et al., 2024). The extraction process using MAE, an eco-friendly and economical method, was optimized using the Box-Behnken design (BBD) statistical method. This approach allows researchers to evaluate multiple factors simultaneously. By combining Response Surface Methodology (RSM) with BBD, experts can establish optimal extraction conditions, ensuring the maximum yield and effectiveness of medicinal plant material extracts (Shabangu et al., 2024). Several studies have demonstrated that RSM can predict the optimal extraction conditions to obtain the highest extraction levels from medicinal plants (Ameer et al., 2022).

*Serjania Erecta* Radlk is traditionally used in folk medicine because of its neuroprotective properties, which may assist in enhancing memory and cognitive function. Similarly, *Xanthoceras sorbifolium*, often referred to as the yellow horn, has improved potential to support neurological health, with some studies suggesting that it may help improve cognitive decline (Mustapha and Taib, 2022; Zang et al., 2021). *In vitro* studies on the aerial parts of *C. halicacabum* have been conducted to treat neurodegenerative disorders (Mahnashi et al., 2021). In silico methods have significantly improved drug discovery by using computational tools to predict interactions between drug candidates and molecular targets. These methods use structure-based approximations and target molecule structures to identify and optimize ligands. Techniques such as molecular docking, molecular dynamics, and density functional theory help identify promising candidates for experimental validation, reducing time and cost. The accuracy of these predictions depends on the target structural data and simulation algorithms employed (Vázquez et al., 2020).

In our previous research, potential Aβ and AchE compounds were reported from extracts of *E. monogynum*, *C. carandas*, and *N. cadamba* (Kareti and Subash, 2020a; Subash and Rao, 2021; Kareti and Subash, 2020b). Tau, a microtubule-associated protein, has been linked to symptom severity in AD, suggesting that targeting tau may be more effective in treating cognitive decline (Congdon et al., 2023; Chen and Yu, 2023). Therapeutically responsive compounds from natural products are gaining popularity over synthetic drugs because of their biocompatibility (Andrade et al., 2023). The essential oil extracted from *Citrus aurantium* leaves using microwave-assisted extraction followed by GC-MS analysis and enzyme inhibition was found to be effective against AD (Elhawary et al., 2024). The main objectives of the present study were (a) to explore and optimize the extraction conditions from the leaves of *C. halicacabum* and identify the phytoconstituents using GC and (b) to utilize *in silico* methods to evaluate anti-AD capabilities against selected targets (Aβ, AChE, and tau) and identify potential lead compounds that may be responsible for these features. The investigated compounds produced encouraging results in *in silico* docking, drug similarity, ADMET prediction, molecular dynamics simulations, and density functional theory (DFT) analysis. These results showed strong pharmacokinetic and pharmacodynamic characteristics, along with acceptable binding energies for the modified derivatives. The inhibitor-surface interaction properties and stability of the docked complex were determined to be sufficient. Overall, these results imply that *C. halicacabum* ligands have considerable potential as effective therapeutic options, warranting further experimental validation.

## 2 Materials and methods

### 2.1 Chemicals

Highly pure ethanol was procured from Loba Chemie Pvt., Ltd.

### 2.2 Collection, identification and drying of plant materials

Leaves of the plant material (*C. halicacabum*) were collected from the Kolli Hills on dry and sunny days. The plant specimens were mounted in a herbarium and identified by Dr. S. P. Subramani Institute of Forest Genetics and Tree Breeding (IFGTB), A voucher was prepared from herbarium specimens (Ref: 25203) stored in the IFGTB (Supplementary Figure S1). The collected leaves were washed and air-dried for 10 days. The dried plant material was finely ground using a homogenizer and stored at room temperature.

### 2.3 Box-Behnken experimental design

The RSM model was created using BBDs because it is more straightforward and effective than alternative three-level factorial designs (Pinlap et al., 2024). The factorial design incorporated three levels of independent variables (powder weight, solvent volume, and extraction time) categorized as −1, 0, and +1, as shown in (Supplementary Table S1). In single-factor analysis, each independent variable was examined separately from the dependent variable. The methodology revealed the contribution of each component to the final result, as shown inthe summary results of the regression analysis in Supplementary Table S2. The response function is more accurate when the center points are included because they account for the possible bias and variability of the setup. The RSM was used to fit the second-order polynomial model equation (Equation 1).

$$Y=b_{0}+b_{1}X_{1}+b_{2}X_{2}+b_{3}X_{3}+b_{12}X_{1}X_{2}+b_{13}X_{1}X_{3}+b_{23}X_{2}X_{3}$$ (1)

where Y represents the observed dependent value for each of the three levels of the factors; X_1_ represents the weight of the powder in grams (g), X_2_ represents the solvent volume in milliliters (mL), and X_3_ represents the extraction time in minutes (min), which are the linear coefficients of the three factors. The dimensionless value of an independent variable, the actual value at its center, and the change in its actual value correspond to changes in its dimensionless value.

### 2.4 Microwave-assisted extraction

MAE uses microwave radiation to heat plant materials and solvents, resulting in the more efficient extraction of valuable compounds. The extraction process was initiated based on previous reports by mixing approximately 1 g of the *C. halicacabum* sample with 15 mL of the selected ethanol-water solution (70:30) in a 100 mL beaker (Boateng, 2023). To enhance the solvent absorption, the mixture was magnetically stirred for 3 minutes, allowing thorough soaking and ensuring that the solvent began to diffuse into the sample matrix. The MAE was performed in a laboratory using a Morphy Richards 28-litre microwave oven capable of delivering a maximum power output of 300 W and operating at a frequency of 2.45 gigahertz. After irradiation, the samples were quickly cooled to 40°C in an ice bath to ensure a rapid temperature reduction. To enhance the purification and precipitate soluble impurities, 6 mL of cold fresh ethanol was added to the filtered sample. The extract was passed through a 0.22 µL membrane filter, and the filtrate was further filtered using Whatman filter paper and purified.

### 2.5 Gas chromatography-mass spectrometry (GC-MS) analysis

GC–MS analysis of the extracted samples was performed using a Shimadzu GCMS-QP2010 Plus instrument featuring a capillary column. The following experimental technique was performed in a stepwise manner at a flow rate of 1.2 mL/min using helium as the carrier gas. Initially, the instrument was set at 50°C and maintained at this temperature for 1.5 min. Subsequently, the temperature was increased to 300°C at a rate of 10°C/min and maintained for 7 min to ensure a comprehensive examination of the compounds present. The temperature was increased to a maximum of 400°C at rate of 4°C/min. Throughout the experiment, the injector and interface temperatures were set to 220°C and 240°C, respectively. These steps are vital for the successful separation and identification of target compounds. The total duration of the mass spectral scan was 30 min, as recognized by the National Institute of Standards and Technology (NIST).

### 2.6 Experimental

#### 2.6.1 Hardware specification

Advanced Micro Devices, Ryzen 5 5500U with a Radeon graphics processor, and Windows 11 were used in this study.

#### 2.6.2 Software specifications

The Box–Behnken design under RSM analysis was performed using Design Expert 12 to optimize the experimental conditions. Ligand structures were generated using ChemDraw 16.0, with subsequent Simplified Molecular Input Line Entry System (SMILES) notations translated via the cactus.nci.nih.gov platform. Molecular docking studies and intermolecular interactions were conducted using the Integrated Graphical Environment for Molecular Docking (iGEMDOCK) V2.1 software and Discovery Studio Visualizer V20. *In silico* ADME-Tox and drug-like predictions were performed using Preadmet. webservice. bmdrc. org and Targetnet. scbdd. com, respectively. The visualization of intermolecular interactions was enhanced using LigPlot plus V2.1. The protein-ligand complexes were solved using chemistry at the Harvard Macromolecular Mechanics-Graphical User Interface (CHARMM-GUI). Molecular Dynamics (MD) simulations were conducted using interfaces with visual Molecular Dynamics (VMD) and nanoscale Molecular Dynamics (NAMD), which are compatible with commonly used quantum mechanics software. Additionally, Density Functional Theory (DFT) calculations were performed using the Gaussian 09 software suite to provide a comprehensive theoretical framework for the study.

### 2.7 Preparation of the receptors

The preparation of receptors using tools such as the Protein Data Bank (PDB) and PyRx is a crucial step in the study of structural targets such as amyloid-β (Aβ), acetylcholinesterase (AChE), and tau filaments (PDB ID: 1IYT, 4EY7, and 5O3L). These tools enable researchers to meticulously remove water molecules and co-crystallized ligands from selected protein structures, thereby ensuring a cleaner and more accurate model for further analysis. Using the vast repository of structural data available in the PDB and the advanced computational capabilities of PyRx, scientists can streamline the receptor preparation process, facilitating more precise docking and molecular dynamics simulations. This refined approach is vital for understanding the interactions and mechanisms underlying neurodegenerative diseases, and ultimately aids in the development of targeted therapeutics (Jabir et al., 2021).

### 2.8 Preparation of ligands

The PDB format is a standardized method for representing the three-dimensional (3D) structure of ligands. Chem3D is a powerful computational chemistry tool that has been employed to optimize the energy of ligands using the Molecular Mechanics 2 (MM2) force field, a method known for its accuracy in modeling small organic molecules. By reducing the energy, this tool helps to determine the most stable configurations of ligands, which is crucial for understanding their behavior and interactions in various chemical environments. This process involves calculating the potential energy surfaces and adjusting the molecular geometries to minimize these energies, leading to more accurate and reliable ligand models in their lowest energy states (Saini et al., 2024).

### 2.9 Molecular docking study

An integrated strategy using iGEMDOCK is critical for the development of effective drug discovery strategies. Rapid virtual screening allows researchers to scan and test a vast array of compounds within a short timeframe. This study used stable standard dock settings with a population size of 200, 70 generations, and two solutions, providing sufficient iterations to refine the results and effectively identify the optimal binding positions. These parameters were used to identify the best ligands based on their conformations and lowest binding free energies. The combination of speed, accuracy, and ease of use creates an environment in which innovative treatments with potential therapeutic efficacy can be developed rapidly (Azad, 2023).

### 2.10 In silico drug-likeness and ADME-Tox prediction

The analysis followed the well-established drug-likeness properties of Lipinski’s Rule of Five (RO5). *In silico* ADME-Tox is a set of criteria used to predict the efficacy of oral compounds in human systems. All nine active ligands (higher docking scores) of *C. halicacabum* and standard galantamine were evaluated using RO5 and ADME-Tox (Daoud et al., 2021). The optimal drug’s 40–130 molecular refractivity (MR) of the drug was <500 Da molecular weight (MW), <5 hydrogen bond acceptors (HBA), <10 hydrogen bond acceptors (HBA), and <5 lipophilicity (LogP) (Durán-Iturbide et al., 2020). To evaluate the drug-likeness of a compound using the PreADMET service, we first ensured that the molecular structure was saved in the correct MOL file format, which is a standard in chemoinformatics for representing molecules with detailed atomic bond and connectivity information. The following pharmacokinetic characteristics were analyzed for ligands with higher docking scores: blood–brain barrier (BBB) permeability, plasma protein binding, water solubility, and carcinogen identification.

### 2.11 LigPlot analysis

LigPlot is an invaluable tool for studying the interactions between ligands and enzymes. The proposed software provides a clear visual representation of the binding modes, key interactions, and spatial arrangements that are critical for understanding these relationships. The plot shows how the ligand fits into the active site of the enzyme, revealing the orientation and position that are essential for its function. LigPlot highlights the various interactions that occur between the ligand and enzyme, including electrostatic interactions, hydrogen bonds, and hydrophobic contacts. LigPlot is a user-friendly and informative tool for analyzing ligand-enzyme interactions. By providing detailed interaction plots, this software not only assists in understanding the binding modes but also empowers researchers to optimize ligands for therapeutic applications (Agrawal et al., 2024).

### 2.12 Molecular dynamics simulation

MD Simulation is an exciting field that allows the study of the behavior of molecules in dynamic environments. In our exploration, the best-docked poses of the ligands were alongside standard results obtained using docking methods to assess their potential as inhibitors of selected targets (Ghahremanian et al., 2022). The NAMD software was designed to efficiently compute large biomolecular systems. This enables researchers to simulate the physical movement of atoms and molecules over time. CHARMM-GUI is an online tool designed to produce crucial ligand topology file. The NAMD input generator optimizes the binding energies of ligands from typical complexes (Thandivel et al., 2024; Kim et al., 2020). The input generator features streamlined the process of preparing simulation files, making it accessible even to users with limited programming experience. The NAMD input generator function of CHARMM-GUI plays a crucial role in the development of ligand topology files. This facilitated the generation of accurate topology and parameter files necessary for simulating dynamic interactions between a ligand and its target protein. By focusing on the ligand with the highest binding energy and its standard complex with the protein, researchers can gain insights into the molecular mechanisms driving this interaction (Vanaja et al., 2025; Feng et al., 2023).

The CHARMM-GUI input generator for NAMD optimized ligand-binding energies by refining the complex structures before the simulation setup. To ensure a biologically relevant environment, the TIP3P water model was employed for explicit solvation to simulate the physiological aqueous conditions for protein-ligand interactions. The Particle Mesh Ewald (PME) method was applied for long-range electrostatics, ensuring the accurate treatment of electrostatic interactions under periodic boundary conditions. Potassium (K^+^) and chloride (Cl^−^) ions were added to neutralize the system and maintain the charge balance (Gee et al., 2024). Energy minimization was performed using the steepest descent algorithm for 1,000 steps to eliminate steric clashes and optimize the initial structure of the system. Equilibration proceeded in two phases: 1) an NVT ensemble (constant number of particles, Volume, and Temperature) for 100 ps to stabilize the temperature and 2) an NPT ensemble (constant number of particles, Pressure, and Temperature) for 100 ps to equilibrate both temperature and pressure. The system was maintained at 310 K using a Langevin thermostat, whereas the pressure was controlled using the Nosé–Hoover Langevin piston method. The production MD simulation was conducted for 50 ns under the NPT ensemble with a 2 fs integration time step. The CHARMM36 force field was employed for both the protein and ligand to ensure an accurate representation of molecular interactions. Long-range electrostatics were treated using the PME method, and SHAKE constraints were applied to the hydrogen atoms, allowing a larger time step for efficient simulations.

### 2.13 Density functional theory (DFT) analysis

DFT analysis is a crucial computational tool in chemistry and materials science for understanding electron behavior in molecules and materials, particularly in phytochemicals. The HOMO is the highest energy level for electrons, whereas the LUMO is the lowest energy level for electron acceptance. This formula was used to calculate the bandgap. All these names originate from quantum mechanics calculations. The HOMO–LUMO energy differential (∆E) (Equation 2), chemical potential ( μ ) (Equation 3), chemical hardness ( η ) (Equation 4), chemical softness ( σ ) (Equation 5), electronegativity ( $\left.X\right)$ ( Equation 6 ), and electrophilicity $\left(\omega \right.$ ) (Equation 7) were determined using the following equations (Mumit et al., 2020).

$$∆E=E_{Homo}-E_{Lumo}$$ (2)

$$\mu =\frac{-\left({E_{Homo}+E}_{Lumo}\right)}{2}$$ (3)

$$\eta =\frac{{E_{Homo}-E}_{Lumo}}{2}$$ (4)

σ=1/η (5)

$$X=\frac{{1+E}_{Lumo}}{2}$$ (6)

$$\omega =\frac{{µ}^{2}}{2\eta }$$ (7)

## 3 Result

### 3.1 Design of experiment

**FIGURE 2:**
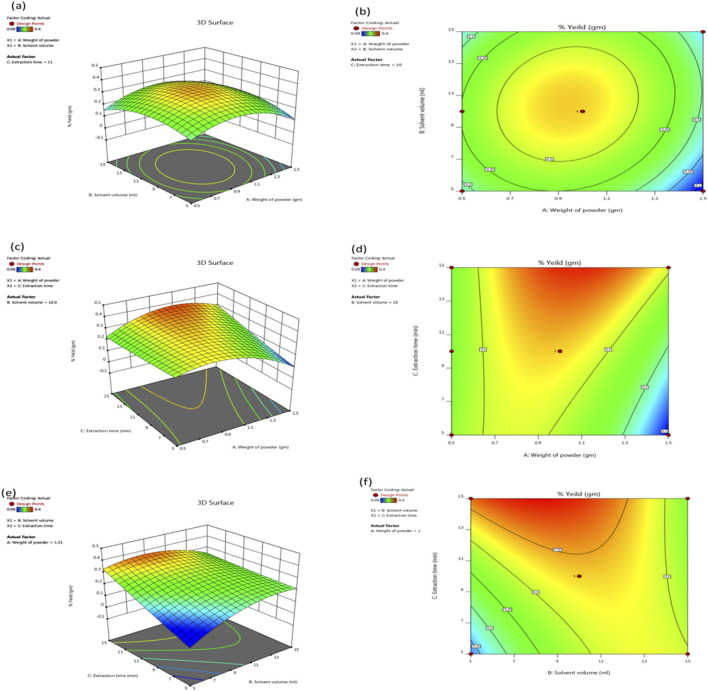
A 3D response surface **(a, c, e)** and 2D contour plots **(b, d, f)** illustrating the effects of solvent volume (15 mL), extraction time (15 min), and weight of the powder (1.5 gm) on the extraction efficiency of *C. halicacabum* are shown in the graphics. These plots show how the yield varies according to the different variables that were tested in the BBD.

BBD is a powerful tool that researchers can use to maximize extraction yields by carefully considering various factors. RSM is a statistical technique used for developing, improving, and optimizing processes and is highly effective for fine-tuning experimental procedures. By employing BBD, a type of experimental design within RSM, researchers can systematically evaluate the interactions between multiple variables with a reduced number of experiments. *C. halicacabum* leaf extract was determined using a statistical approach, specifically the BBD. The proposed method maximizes extraction yield by systematically varying these three factors. In this study, we determined three main parameters: the powder weight (1.5 g), solvent volume (15 mL), and extraction duration (15 min). A weight ratio of 0.4% emerged as a significant indicator for detecting variations in yield under optimal extraction conditions that maximized the yield and quality of the extract while minimizing resource use and time. Figure 2 illustrates our response surface technique, and the data presented in this way help us understand the underlying relationships between the variables.

### 3.2 Compound identification by GC-MS

**FIGURE 3:**
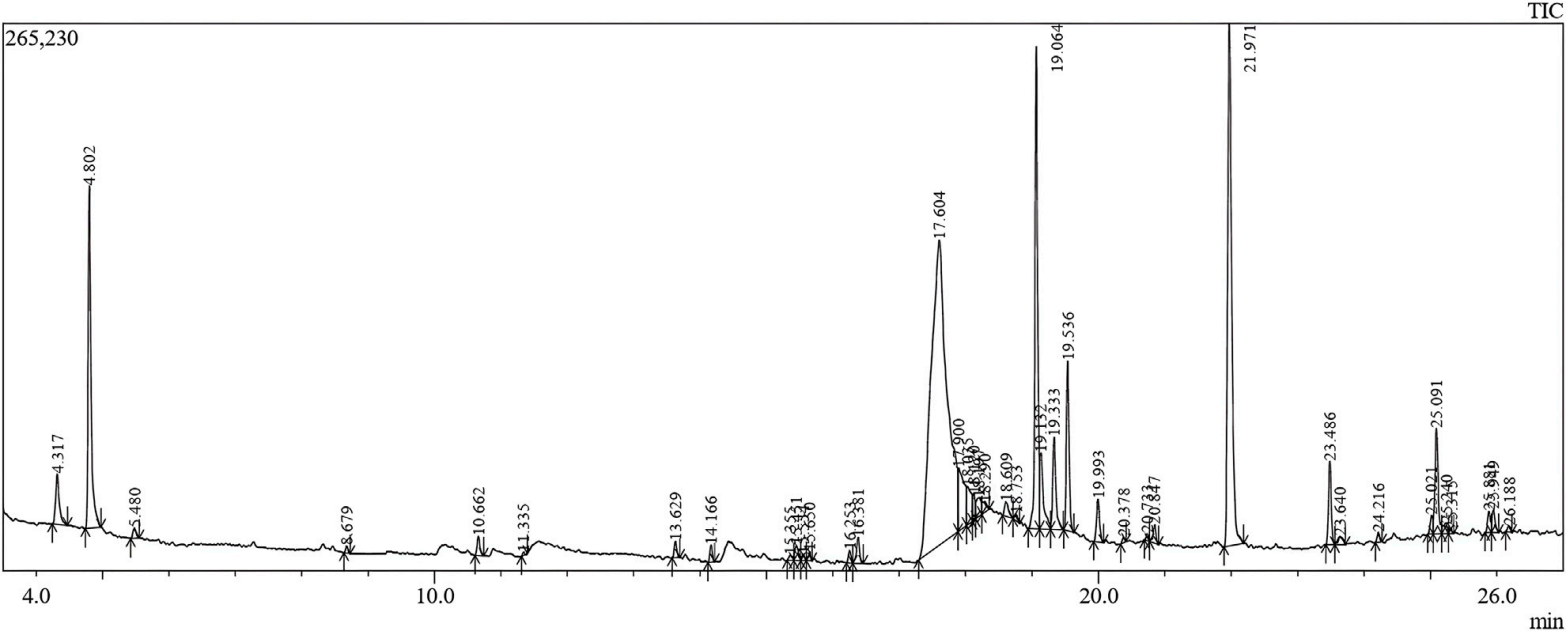
GC–MS chromatogram of microwave assisted extract of *C. halicacabum* leaf.

GC-MS is an efficient method for studying complex mixtures and identifying individual compounds in the extracts. The system separates the samples, identifies them based on their mass spectra, and identifies precise phytochemicals. This technique is not only reliable but also capable of analyzing complex mixtures with a high degree of sensitivity. In this study, 40 phytoconstituents were identified using GC–MS analysis of an ethanolic extract of *C. halicacabum* leaves and a GC-MS chromatogram (Figure 3); Table 1 shows the identified phytoconstituents, which were aligned based on their retention times (RT) in *C. halicacabum* leaf extract. The retention time and mass spectra associated with each peak provide detailed information regarding the molecular structure and quantity of each constituent.

**TABLE 1:** The binding energy between the selected AD targets and the ligands (phytoconstituents) identified from *C. halicacabum* leaves.

S. No	Retention time	Peak area (%)	Name of the ligands (Identified by the GCMS)	Binding energy (Kcal/mol)		
				1IYT	4EY7	5O3L
1.	4.317	1.44	1,1-Diethoxy-2-butene	−272.03	−56.7974	−55.6313
2.	4.802	7.80	2-Methyl-1,3-dioxolan-4-one	−267.553	−55.8781	−50.126
3.	5.480	0.25	Hydroperoxide, 1-methylethyl	−247.091	−45.3799	−43.3169
4.	8.679	0.12	Pentane, 1,5-dibromo	−199.834	−42.5457	−51.8451
5.	10.662	0.41	Cyclododecanone	−214.499	−67.5996	−67.8949
6.	11.335	0.04	Cyclopropane	−109.367	−30.5775	−24.2281
7.	13.629	0.32	3-Hexanone, 2,2-dimethyl-	−249.975	−51.7541	−53.7594
8.	14.166	0.32	Methacryloyl chloride	−223.367	−42.8274	−43.3823
9.	15.355	0.14	(2E)-2-Butenoyl Chloride	−226.535	−41.5719	−43.2975
10.	15.451	0.41	Vinyl (2E)-2-butenoate	−265.006	−53.1716	−57.7498
11.	15.554	0.12	2-Nitro-1-Octanol	−243.124	−72.9643	−61.8083
12.	15.650	0.15	4-Allyl-2-T-Butyl-3-(naphthalene-2-carbonyl)oxazolidin-5-One	−326.797	−84.9845	−80.5331
13.	16.253	0.25	3-Hexanone, 2,2-Dimethyl-	−256.675	−51.9641	−52.1458
14.	16.381	0.61	(−)-5-Oxatricyclo[8.2.0.0(4,6)]dodecane,12-trimethyl-9-methylene-, [1R-(1R*,4R*,6R*,10S*)]-	−228.29	−71.4298	−73.6642
15.	17.604	38.14	Methyl hexofuranoside	−366.639	−79.4068	−79.5866
16.	17.900	2.93	1,3-Dioxane, 2-(1,3-Dioxolan-2-Yl)-	−239.827	−63.4689	−67.7471
17.	18.035	1.26	(Z)-6-(tetrahydropyran-2-yloxy)-1-trimethylsilylhex-2-ene	−265.073	−77.0876	−78.9575
18.	18.120	0.52	Benzene, pentachloro(trichloroethenyl)	−217.618	−73.2043	−61.8653
19.	18.197	0.72	Propane, 2-fluoro-2-methyl	−133.917	−36.2238	−32.2317
20.	18.290	0.33	Propane, 2-methoxy-2-methyl	−223.73	−42.4179	−38.3854
21.	18.609	0.43	Tetradecane, 2-methyl	−243.54	−76.3938	−105.444
22.	18.753	0.15	(1e)-1-Nitro-1-propene	−280.004	−53.2683	−50.0026
23.	19.064	10.32	Neophytadiene	−269.455	−79.9903	−90.6412
24.	19.132	2.08	2-Undecene, 9-methyl-, (Z)	−235.084	−59.3566	−67.9578
25.	19.330 19.536	5.84	3,7,11,15-Tetramethyl-2-hexadecen-1-ol	−293.78	−74.78	−83.5375
26.	19.993	1.01	D-Norleucine	−264.096	−53.0345	−56.1045
27.	20.378	0.09	Butanoic acid	−240.334	−39.215	−51.5357
28.	20.733	0.13	Oxalic acid, dineopentyl ester	−290.206	−74.9915	−81.7085
29.	20.847	0.40	Methacrylic anhydride	−310.705	−61.7779	−62.9562
30.	21.971	16.55	Phytol	−344.749	−75.8905	−103.477
31.	23.486	1.72	Bis(2-(Dimethylamino)ethyl) ether	−222.787	−61.515	−69.3703
32.	23.640	0.30	1-(2-Hydroxyethoxy)-2-methyldodecane	−304.794	−67.8612	−107.91
33.	24.216	0.19	8-Methyl-7-nonen-5-olide	−272.76	−66.695	−73.936
34.	25.021	0.39	3-Methoxysulpholane	−255.088	−57.2789	−61.7895
35.	25.091	2.53	3-Cyclopentylpropionic acid, 2-dimethylaminoethyl ester	−267.129	−77.4302	−77.1911
36.	25.240	0.36	Acetic acid, (dodecahydro-7-hydroxy-1,4b,8,8-tetramethyl-10-oxo-2(1h)-phenanthrenylidene)-, 2-(dimethylamino)ethyl ester, [1R-(1.alpha)]	−370.848	−97.2995	−92.278
37.	25.315	0.11	Methoxyacetic acid, 2-tetrahydrofurylmethyl ester	−285.693	−64.7099	−69.8258
38.	25.881	0.47	1,2-benzenedicarboxylic acid, diisooctyl Ester	−340.699	−87.3986	−80.9808
39.	25.949	0.54	N-(2,6-dimethyl-phenyl)-N-(2-morpholin-4-Yl-2-phenyl-acetyl)-benzamide	−314.705	−87.7163	−87.1485
40.	26.188	0.13	2(3H)-Furanone, dihydro-3-hydroxy-4,4-dimethyl	−259.935	−59.374	−61.5798
Std	-		Galantamine	−363.895	−84.4037	−85.5439

### 3.3 Identification of new compounds from C. halicacabum

By comparing the *C. halicacabum* leaf extract with previously reported compounds (Dowlath et al., 2020; Jeyadevi et al., 2013; Saikia et al., 2023; Karuppannan et al., 2020; Bhagat and Bhuktar, 2020; Divya et al., 2018), GC-MS analysis revealed 40 phytoconstituents (Table 1). A comparison of reported phytoconstituents is presented in Supplementary Table S3. Among these, 37 compounds were newly identified, and three compounds have been previously reported in the literature for *C. halicacabum* leaves: 3,7,11,15-tetramethyl-2-hexadecen-1-ol, phytol, and neophytadiene. A detailed list of phytoconstituents from *the C. halicacabum* leaf extract provides essential information for researchers to reference and compare these compounds for further studies and applications.

### 3.4 Ligand preparation

**FIGURE 4:**
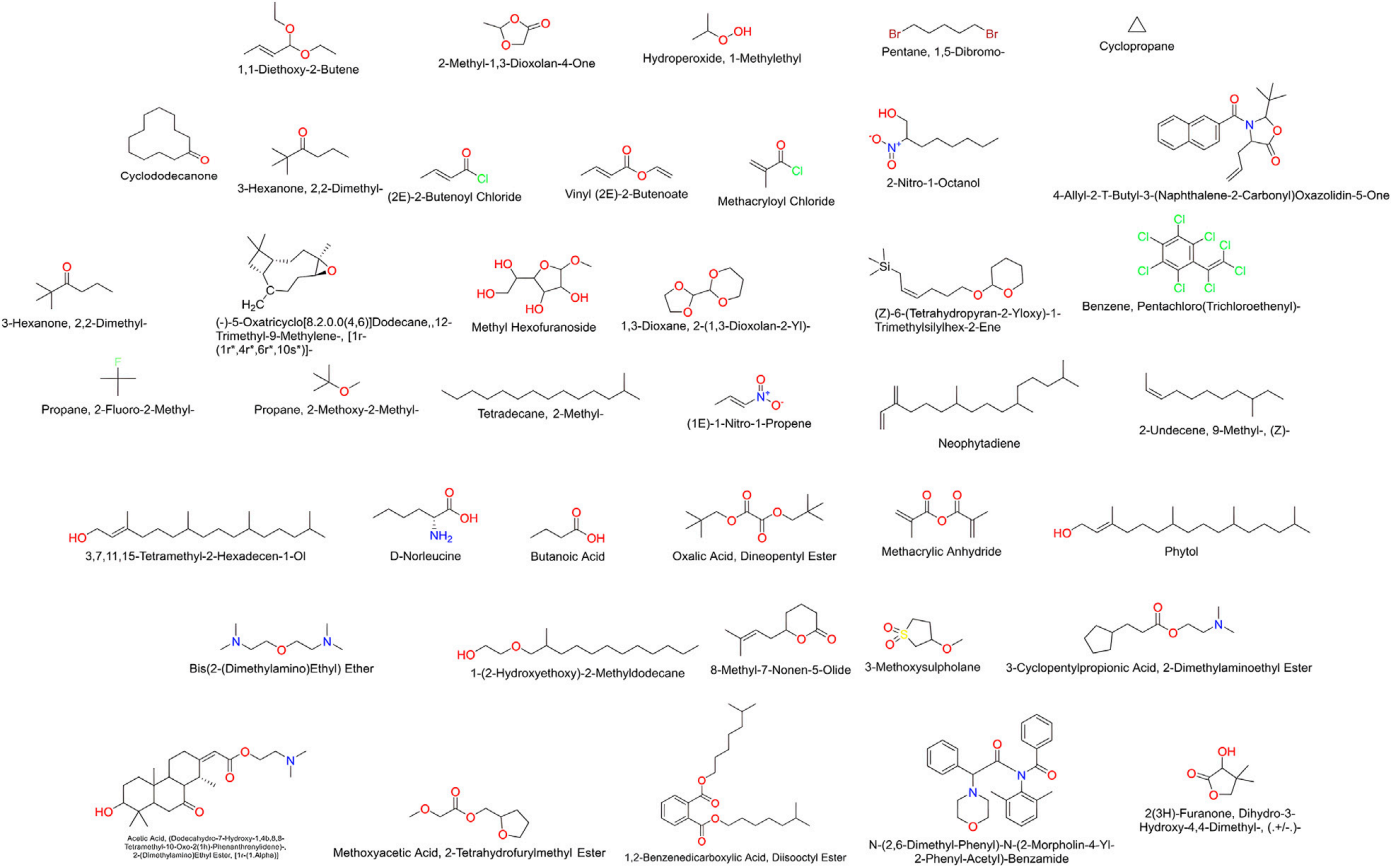
Compounds detected in extract of *C. halicacabum* leaf by GC-MS.

The ligands identified by GC-MS from the *C. halicacabum* extract are shown in Figure 4. For a comprehensive overview, a complete list of these ligands is presented in Table 1. This structured presentation facilitates easy reference and comparison, facilitating further studies and applications of the *C. halicacabum* extract.

### 3.5 Molecular docking

The best-pose ligands for AD targets were identified in this study. Compounds with the highest binding affinities to receptors were selected as possible lead compounds. Compounds with anti-Alzheimer’s potential (lowest binding energy) in the *C. halicacabum* leaf extract were confirmed using iGEMDOCK. Based on their strong binding affinities to their targets, all 40 phytoconstituents were evaluated against three AD receptors (Aβ - 1IYT; AChE - 4EY7; and Tau - 5O3L). The binding energies of the selected AD targets and their ligand interactions are summarized in Table 1.

#### 3.5.1 Top dock score phytoconstituents interaction with 1IYT

The galantamine standard (−363.895 kcal/mol) was compared with two phytoconstituents that showed the lowest binding energy and highest docking score: methyl hexofuranoside (−366.639 kcal/mol), acetic acid (dodecahydro-7-hydroxy-1,4b,8,8-tetramethyl-10-oxo-2(1H)-phenanthrenylidene), and 2-(dimethylamino)ethyl ester (−370.848 kcal/mol).

#### 3.5.2 Top dock score phytoconstituents interaction with 4EY7

The galantamine standard (−84.4037 kcal/mol) was compared with four phytoconstituents that showed the lowest binding energies and highest docking scores: 4-allyl-2-t-butyl-3-(naphthalene-2-carbonyl)oxazolidin-5-one (−84.9845 kcal/mol), acetic acid (dodecahydro-7-hydroxy-1,4b,8,8-tetramethyl-10-oxo-2(1H)-phenanthrenylidene)-, 2-(dimethylamino)ethyl ester, [1R-(1. alpha (−97.2995), 1,2-benzenedicarboxylic acid, diisooctyl ester (−87.3986), N-(2,6-dimethyl-phenyl)-N-(2-morpholin-4-yl-2-phenyl-acetyl)-benzamide (−87.7163).

#### 3.5.3 Top dock score phytoconstituents interaction with 5O3L

The galantamine standard (−85.5439 kcal/mol) was compared with six phytoconstituents that showed the lowest binding energies and highest docking scores: tetradecane, 2-methyl-(−105.444); neophytadiene (−90.6412), phytol (−103.477), 1-(2-hydroxyethoxy)-2-methyldodecane (−107.91), acetic acid (dodecahydro-7-hydroxy-1,4b,8,8-tetramethyl-10-oxo-2(1H)-phenanthrenylidene)-, 2-(dimethylamino)ethyl ester (−92.278), and N-(2,6-dimethyl-phenyl)-n-(2-morpholin-4-yl-2-phenyl-acetyl)-benzamide (−87.1485).

Common lead phytoconstituents include acetic acid (dodecahydro-7-hydroxy-1,4b,8,8-tetramethyl-10-oxo-2(1H)-phenanthrenylidene) and 2-(dimethylamino)ethyl ester (1R- [1. alpha)]. Additionally, for the specific targets 4EY7 and 5O3L, N-(2,6-dimethyl-phenyl)-N-(2-morpholin-4-Yl-2-phenyl-acetyl)-benzamide was identified as the common lead. The docking results suggested that these nine phytoconstituents underwent *in silico* drug likeness and ADME-Tox prediction.

### 3.6 Prediction of binding sites

**FIGURE 5:**
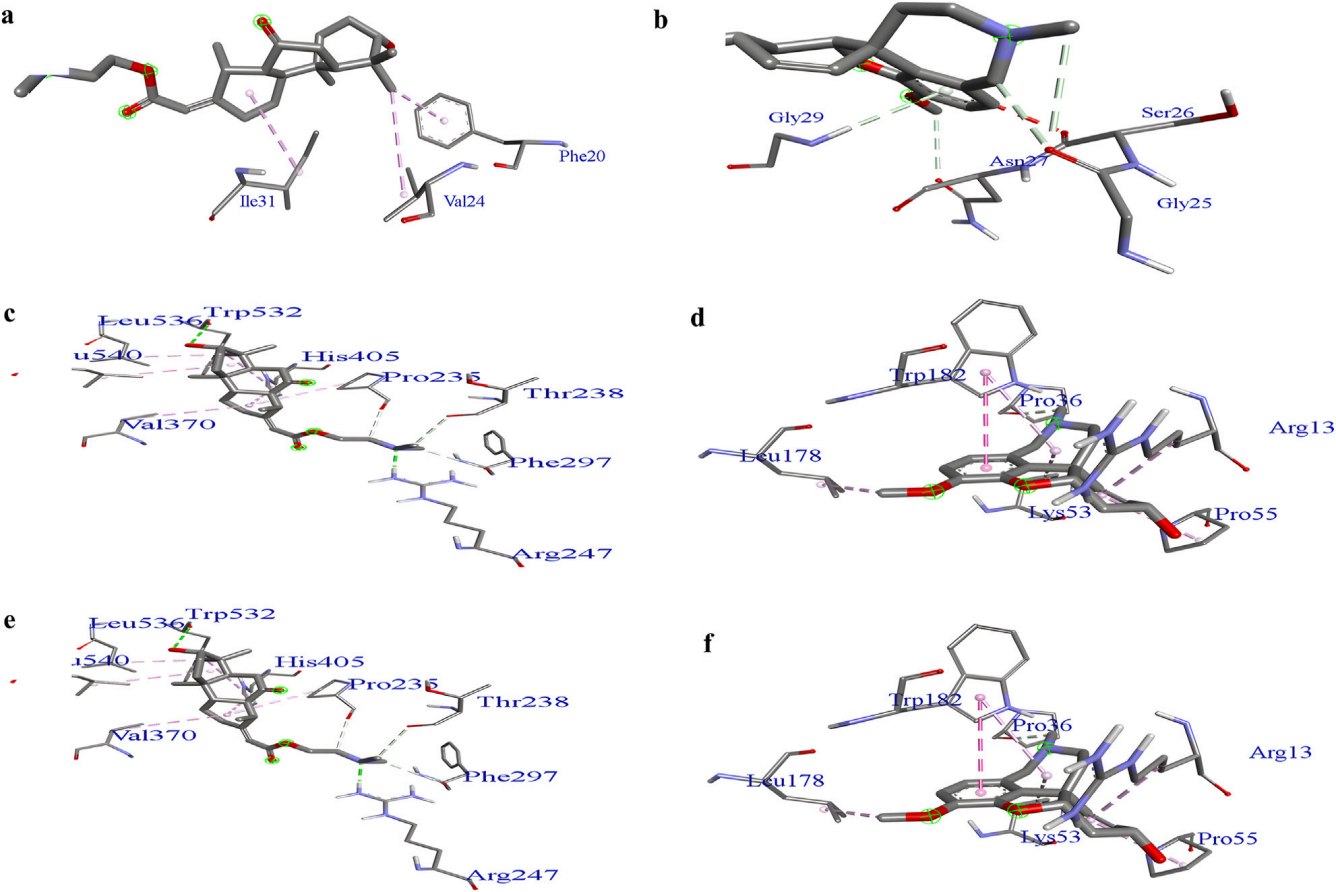
Binding pockets: 1IYT with ligand 35 **(a)**, 1IYT with galantamine **(b)**, 4EY7 with ligand 35 **(c)**, 4EY7 with galantamine **(d)**, 5O3L with ligand 31 **(e)** and 5O3L with galantamine **(f)**.

Supplementary Figure S2 illustrates the hierarchical clustering results from the iGEMDOCK post-screening analysis, which highlights the interaction patterns between AD targets and plant components derived from *C. halicacabum* extracts and standards. This clustering analysis helps visualize the similarities and differences in binding interactions, providing insights into the potential efficacy and mechanisms of plant-derived compounds in modulating AD-related biological pathways. Groups with similar interaction patterns offer a clearer understanding of the components that may be most promising for further investigation of AD treatment. Figure 5 Binding pockets of lead compounds and standard complexes with proteins 1IYT, 4EY7, and 5O3L. Discovery Studio Visualizer software was used to predict binding sites in the 1IYT, 4EY7, and 5O3L protein receptors with the lowest binding energy, resulting in higher docking scores. Lower binding energy values typically correlate with higher binding affinities, indicating that ligand–protein interactions are more favorable and stable. Consequently, sites with the lowest binding energies can result in higher docking scores, providing valuable insights into the development of effective inhibitors or therapeutic agents that target these proteins.

### 3.7 *In silico* drug-likeness and ADME-Tox prediction

In recent docking research, nine ligands have emerged as promising candidates for drug-likeness, based on a comprehensive analysis of several critical factors. These ligands were evaluated for their partition coefficient, which indicates their ability to cross cell membranes, and molecular weight, ensuring that they fall within a range suitable for biological activity. Molar refractivity was also assessed to determine the volume and polarizability of the ligands, which influence their interactions with biological targets. Additionally, the number of hydrogen bond donors and acceptors was scrutinized because these properties play crucial roles in the ligand-binding affinity and specificity. Ligands with optimal pharmacokinetic and pharmacodynamic profiles have paved the way for the development of effective and safe therapeutic agents such as i) 4-allyl-2-T-butyl-3-(naphthalene-2-carbonyl)oxazolidin-5-one; ii) methyl hexofuranoside; iii) tetradecane, 2-methyl-; iv) neophytadiene; v) phytol; vi)1-(2-hydroxyethoxy)-2-methyldodecane; vii) acetic acid,(dodecahydro-7-hydroxy-1,4b,8,8-tetramethyl-10-oxo-2(1H)-phenanthrenylidene)-, 2-(dimethylamino)ethyl ester, [1R-(1. alpha; viii)1,2-benzenedicarboxylic acid, diisooctyl ester and ix) N-(2,6-dimethyl-phenyl)-N-(2-morpholin-4-Yl-2-phenyl-acetyl)-benzamide. Furthermore, these compounds exhibited improved ADMET characteristics compared with galantamine (Table 2. All nine phytoconstituents were subsequently analyzed, and drug-likeness and ADMET analyses were performed. All these compounds have shown more than 80% drug-likeness, better blood-brain permeation, high plasma protein-binding capacity, high water solubility, and no carcinogenicity. The phytoconstituents of *C. halicacabum* leaves influence the pathways involved in AD targets.

**TABLE 2:** Drug likeness and ADMET characteristic of the chosen top ligands through the docking analysis.

Ligand No	Drug likeness						ADME-toxicity profile			
	MR	MW	HBD	HBA	LogP	RO5 (%)	BBB	PPB (%)	WS	Carcinogen
	40–130	<500	<5	<10	<5				(mg/L)	
Top ligands for 1IYT										
36	113.216	405	1	5	3.456	100	+	0.719	−3.473	−ve
15	40.776	194	4	6	−2.567	100	+	0.354	0.693	−ve
Top ligands for 4EY7										
36	113.216	405	1	5	3.456	100	+	0.719	−3.473	−ve
39	125.749	428	0	5	4.550	100	+	1.026	−2.353	−ve
38	112.685	390	0	4	6.200	80	+	0.881	−6.578	−ve
12	98.054	337	0	4	4.155	100	+	1.177	−3.93	−ve
Top ligands for 5O3L										
32	74.295	244	1	2	4.162	100	+	0.888	−0.9	−ve
21	71.298	212	0	0	5.953	80	+	0.916	−5.764	−ve
30	95.561	296	1	1	6.364	80	+	0.756	−2.472	−ve
36	113.216	405	1	5	3.456	100	+	0.719	−3.473	−ve
23	94.055	278	0	0	7.167	80	+	0.77	−5.255	−ve
39	125.749	428	0	5	4.550	100	+	1.026	−2.353	−ve
Standard Galantamine										
Std	78.869	287	1	4	1.617	100	+	0.526	−2.53	−ve

### 3.8 LigPlot analysis

**FIGURE 6:**
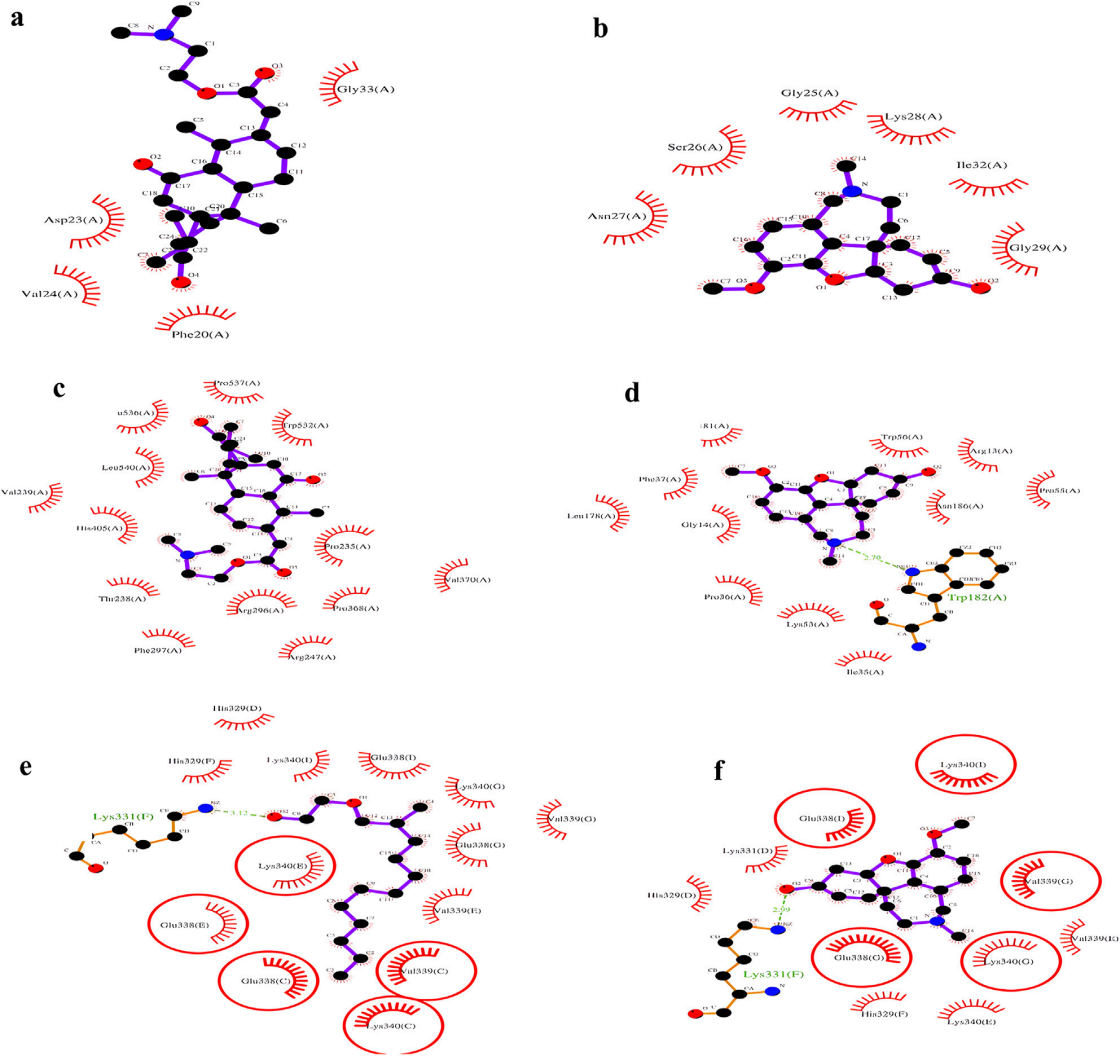
LigPlot analysis: 1IYT with ligand 35 **(a)**, 1IYT with galantamine **(b)**, 4EY7 with ligand 35 **(c)**, 4EY7 with galantamine **(d)**, 5O3L with ligand 31 **(e)** and 5O3L with galantamine **(f)**.

Compounds demonstrating the strongest binding affinity through *in silico* analysis (lead-hit ligand) were subjected to LigPlot analysis to evaluate hydrogen bonding and hydrophobic interactions. The comparison results between the standards and lead-hit ligands are presented in Table 3 and Figure 6. The acetic acid ligand, 2-(dimethylamino)ethyl ester with 1IYT (dodecahydro-7-hydroxy-1,4b,8,8-tetramethyl-10-oxo-2(1H)-phenanthrenylidene)-, exhibited four hydrophobic contacts, but no H-bond interactions were observed. Similarly, 4EY7 exhibited no H-bonds and only 13 hydrophobic interactions were observed when the same ligand was used. There were 13 hydrophobic interactions and one H-bond interaction in 1-(2-hydroxyethoxy)-2-methyldodecane with the 5O3L interaction. Five common binding sites were observed between the ligand 1-(2-hydroxyethoxy)-2-methyldodecane and the standard galantamine: Val339(C), Lys340(C), Glu338(C), Glu338(E), and Lys340(E).

**TABLE 3:** Liplot analysis: hydrogen-bonding and hydrophobic interactions of lead hit ligands and standard complex.

S. No	Complex	Ligplot analysis		
		H-bond interactions		Hydrophobic contacts
		Amino acid	Distance (Å)	Amino acid
1.	1IYT with ligand 36	-	-	Gly33(A), Phe20(A), Val24(A), Asp23(A)
2.	1IYT with standard	-	-	Asn27(A), Gly29(A), Ile32(A), Ser26(A), Gly25(A), Lys28(A)
3.	4EY7 with ligand 36	-	-	Phe297(A), Thr238(A), His405(A), Val239(A), Leu540(A), Leu536(A), Pro537(A), Trp532(A), Pro235(A), Val370(A), Pro368(A), Arg247(A), Arg296(A)
4.	4EY7 with standard	Trp182(A)	2.70	Gln181(A), Phe37(A), Leu178(A), Gly14(A), Ile35(A), Lys53(A), Pro36(A), Asn186(A), Trp56(A), Pro55(A), Arg13(A)
5.	5O3L with ligand 32	Lys331(F)	3.12	His329(D), Lys340(I), Glu338(I), His329(F), Lys340(G), Val329(G), Glu338(G), Val339(E), Val339(C), Lys340(C), Glu338(E), Glu338(C), Lys340(E)
6.	5O3L with standard	Lys331(F)	2.99	Lys340(I), Glu338(I), Lys331(D), His329(D), His329(F), Lys340(E), Lys340(G), Glu338(G), Val339(G), Val339(E)

### 3.9 MD simulation

**FIGURE 7:**
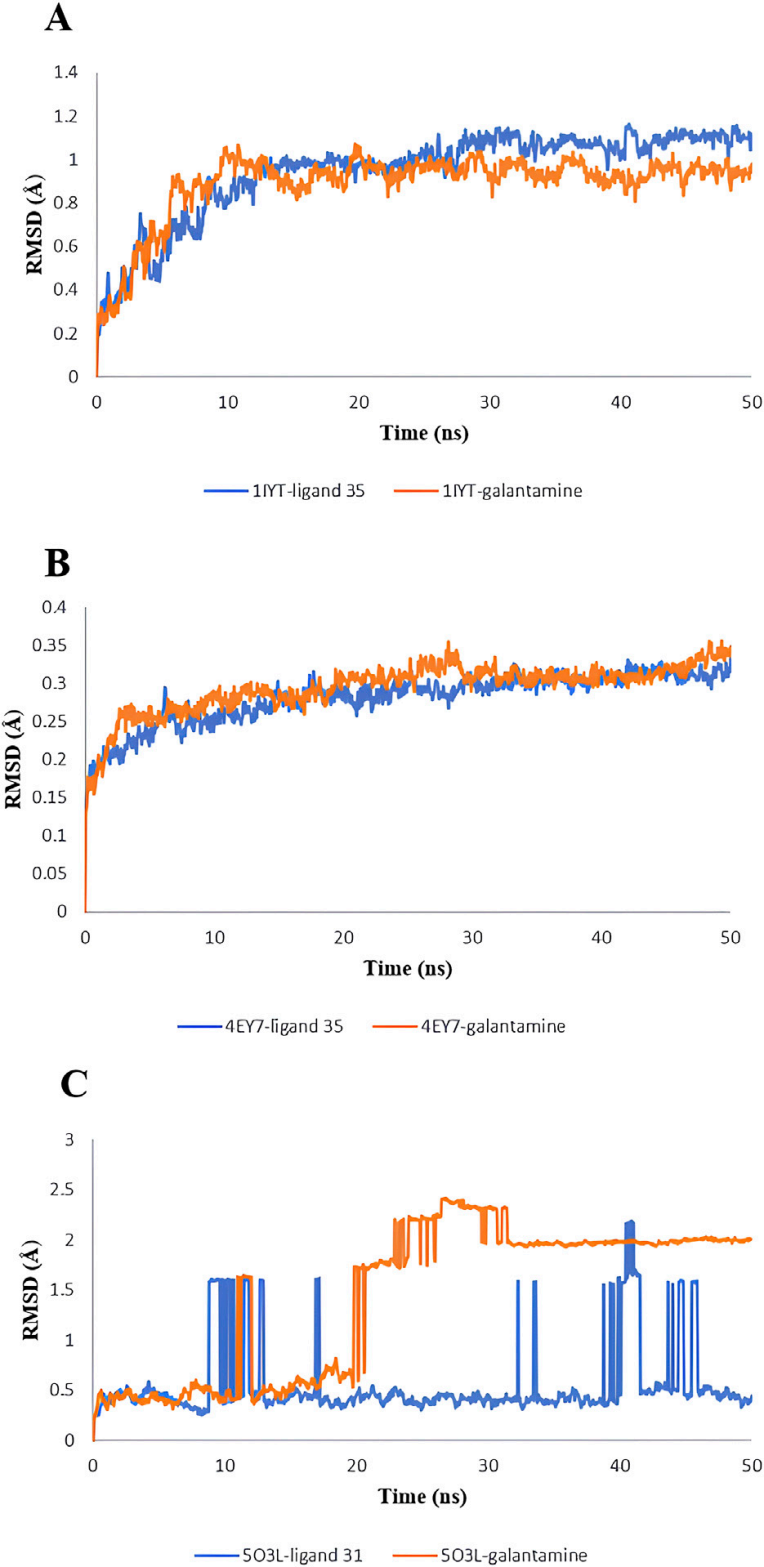
RMSD value: 1IYT with ligand 35 and 1IYT with galantamine **(A)**, 4EY7 with ligand and 4EY7 with galantamine **(B)**, 5O3L with ligand 31 and 5O3L with galantamine **(C)**.

**FIGURE 8:**
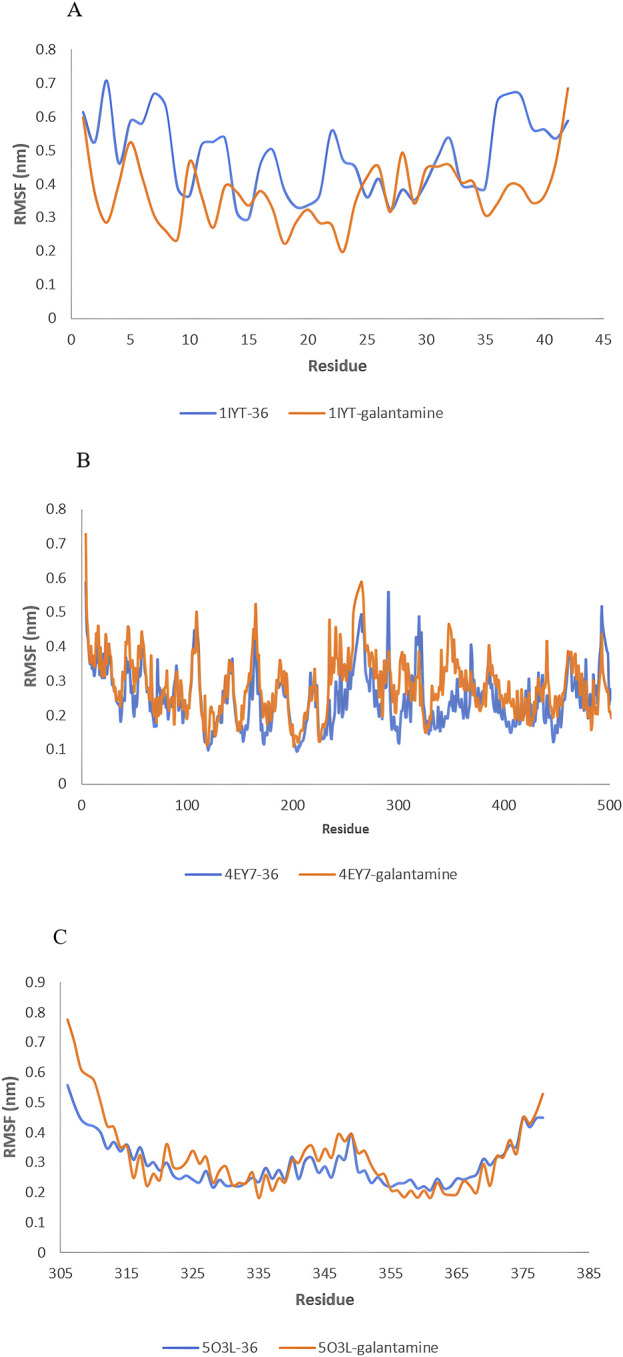
RMSF value: 1IYT with ligand 35 and 1IYT with galantamine **(A)**, 4EY7 with ligand and 4EY7 with galantamine **(B)**, 5O3L with ligand 31 and 5O3L with galantamine **(C)**.

MD simulations were used to identify the protein-ligand complexes with the highest lead hits for the selected target complexes (1IYT-ligand 36, 4EY7-ligand 36, 5O3L-ligand 32, and the standard complex of all targets). The expected affinities of the binding sites of 1IYT, 4EY7, and 5O3L determine their stability. The structural and dynamic features of the protein-ligand complex systems, such as backbone root-mean-square deviation (RMSD) and root-mean-square fluctuation (RMSF), were the major focus of the MD simulation, which studied the behavior of receptors and ligands for 50 ns during the ligand-complex formation phase. RMSD analysis was used to track structural and conformational changes in the protein-ligand complex backbone atoms. The average and standard deviation values are listed in Table 4. For a 50-ns trajectory, the RMSD and RMSF plots of protein-ligand complexes are presented in (Figures 7, 8) were respectively, for each complex to assess their stability. The results revealed that 4EY7 complexed with ligand 36 exhibited the lowest average RMSD (0.281 nm), indicating the highest stability among the complexes studied. In contrast, the 5O3L protein complexed with ligand 32 showed an average RMSD value of 0.552 nm, which was significantly higher but still better than the standard galantamine complex values of 0.294 nm and 1.429 nm, respectively, for the same proteins. These findings suggest that ligand 36 forms a more stable interaction with the 4EY7 protein than the other complexes, potentially making it a more promising candidate for further investigation. 1IYT with ligand 36 (0.944 nm) showed the highest RMSD value compared to that of the standard galantamine complex (0.886 nm), which was also acceptable. A smaller RMSD value implies a greater complex stability. For example, the complex formed between 4EY7 and ligand 36 exhibited a lower RMSD of 0.281 nm than the standard value of 0.294 nm, suggesting that ligand 36 interacts more stably with the receptor.

**TABLE 4:** The average values of RMSD and RMSF of lead hit from *C. halicacabum* and standard from the MD simulation.

S. No	Complex	Average ± SD RMSD (nm)	Average ± SD RMSF (nm)
1.	1IYT with ligand 36	0.944 ± 0.205	0.481 ± 0.114
2.	1IYT with standard	0.886 ± 0.164	0.374 ± 0.096
3.	4EY7 with ligand 36	0.281 ± 0.034	0.250 ± 0.081
4.	4EY7 with standard	0.294 ± 0.034	0.284 ± 0.082
5.	5O3L with ligand 32	0.552 ± 0.388	0.295 ± 0.077
6.	5O3L with standard	1.429 ± 0.755	0.317 ± 0.122

RMSF is a critical parameter in MD simulations that provides insight into the flexibility and stability of protein residues. The 1IYT complex with the standard exhibited a lower RMSF (0.374 nm) than that of its ligand counterpart (0.481 nm), suggesting that it was more stable. Similarly, 4EY7 with ligand 36 showed a lower RMSF (0.250 nm) than that of the standard (0.284 nm), indicating that the ligand contributed to increased stability. However, the 5O3L complex exhibited a higher RMSF (0.317 nm) than the ligand (0.295 nm), which may imply greater flexibility and lower stability than those of the standard galantamine.

**FIGURE 9:**
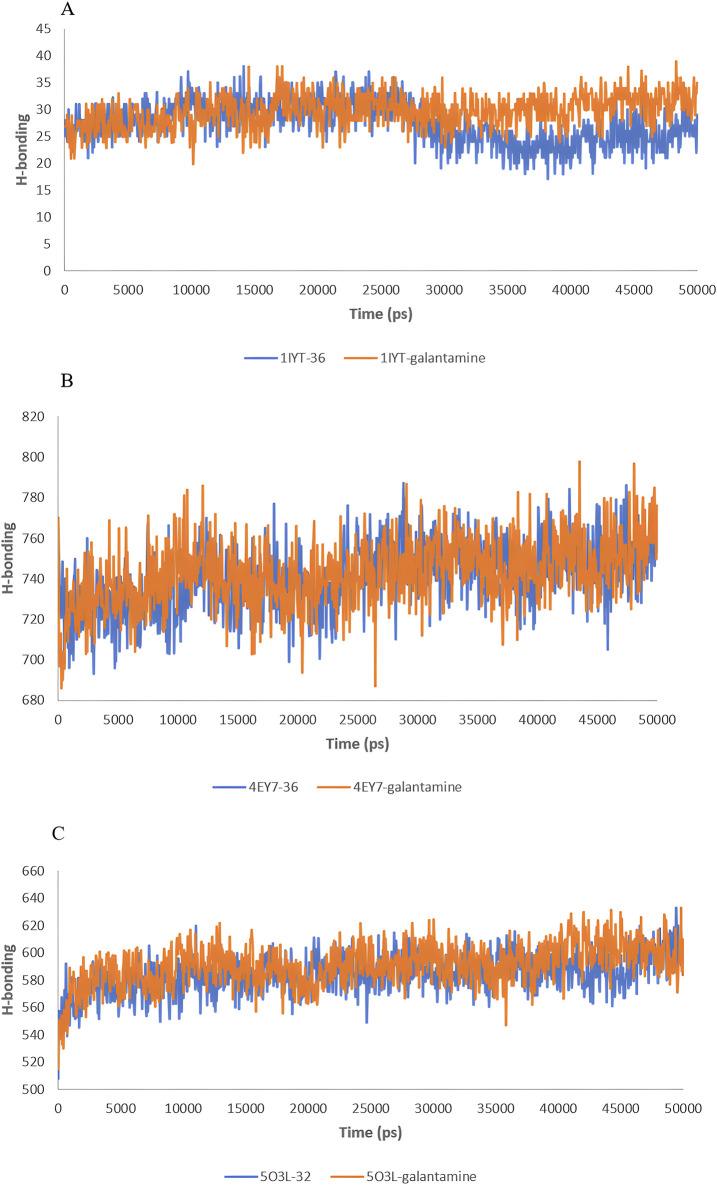
H-bond analysis during 50 ns of the MD simulation: 1IYT with ligand 35 and 1IYT with galantamine **(A)**, 4EY7 with ligand and 4EY7 with galantamine **(B)**, 5O3L with ligand 31 and 5O3L with galantamine **(C)**.

Hydrogen bonds play crucial roles in the stability and functionality of biological molecules, particularly in protein-ligand interactions. The analysis of hydrogen bonding interactions in molecular simulations offers valuable insights into the binding strength and specificity of complexes, which are crucial for drug design and understanding biochemical pathways. The H-bond analysis results of the MD simulations for 50 ns are shown in Figure 9.

**FIGURE 10:**
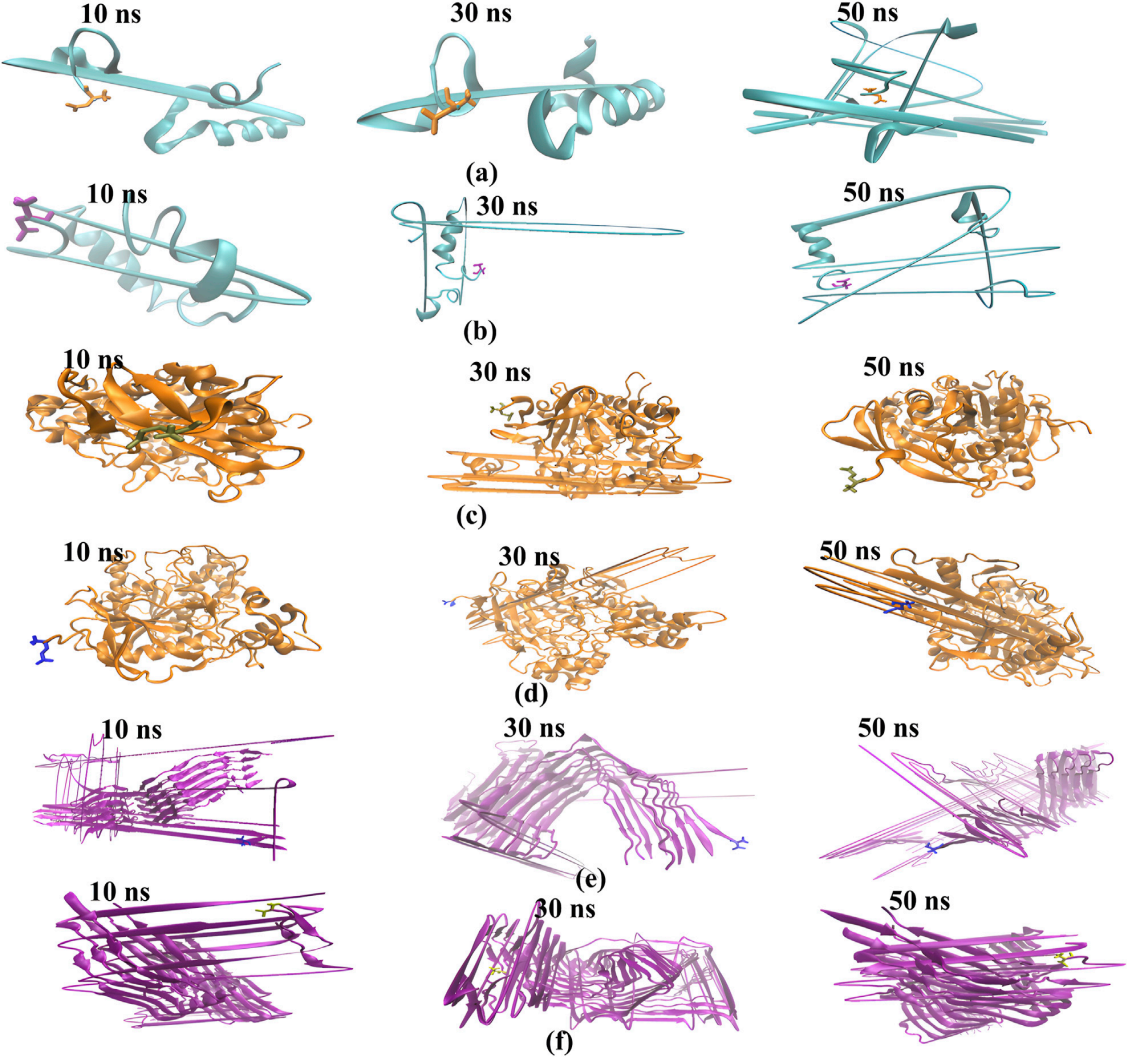
The binding modes of the ligand-protein complexes were captured in snapshots at various intervals (time step = 10, 30, and 50 ns) during the duration of the MD simulation, which lasted for 30 seconds. The cartoon representation shows, 1IYT with ligand 35 **(a)**, 1IYT with galantamine **(b)**, 4EY7 with ligand 35 **(c)**, 4EY7 with galantamine **(d)**, 5O3L with ligand 31 **(e)** and 5O3L with galantamine **(f)**.

For 1IYT-ligand 36 and 1IYT-galantamine, the graph indicates that both exhibit fluctuations in the number of hydrogen bonds over time. However, 1IYT-galantamine consistently demonstrated a higher average number of hydrogen bonds, particularly after the 30 ns mark, at which point the difference became more distinct. Both the 4EY7-ligand 36 and 4EY7-galantamine complexes exhibited a similar trend of increasing hydrogen bonding values over a 50-ns period. Despite experiencing fluctuations, the H-bonding values of these systems generally fell between 700 and 800. These fluctuations are characterized by occasional peaks and troughs, reflecting short-term variations in bonding interactions. Notably, the 4EY7-galantamine complex exhibited slightly higher and more frequent hydrogen bonding peaks than 4EY7-ligand 36, suggesting a marginally stronger or more stable interaction pattern. Finally, the comparison between the 5O3L-ligand 32 and 5O3L-galantamine complexes highlights subtle differences in their hydrogen bonding behaviors over a 50-ns simulation. Both systems initially showed an increase in H-bonding values but eventually stabilized between 560 and 620 bonds. Fluctuations in H-bonding were observed throughout the simulations. However, 5O3L-galantamine consistently exhibited slightly higher H-bonding values, particularly in the latter half of the reaction. This trend suggests that 5O3L-galantamine may form stronger and more stable interactions than the 5O3L-ligand 32. Figure 10.

Snapshots of the dynamic interactions between the ligand and protein complexes in a 50-ns molecular MD simulation. By observing these interactions at intervals of 10, 30, and 50 ns, we gained insight into how the ligand binds and influences the protein’s conformational changes over time. The 10-ns snapshots reveal the initial binding modes and possible rapid adjustments of the complex. At 30 ns, the interactions may exhibit stabilization patterns or transient states that precede the final configuration. Finally, the 50-ns snapshots provide a glimpse into the long-term behavior and stability of the ligand within the binding site, providing a comprehensive view of the interaction dynamics throughout the simulation.

### 3.10 Density functional theory (DFT) analysis

**FIGURE 11:**
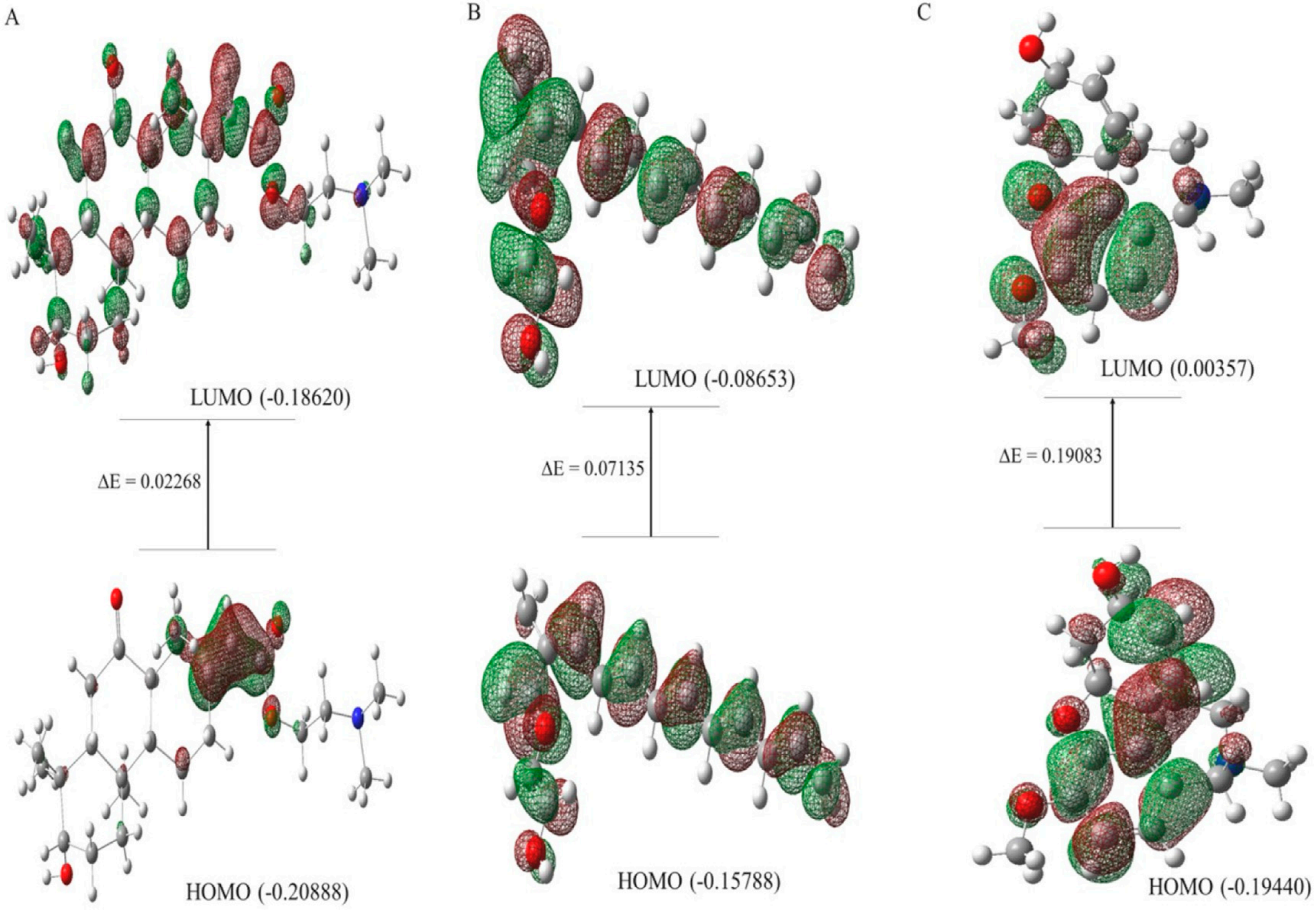
HOMO-LUMO molecular orbital diagram of ligand 36 **(A)**, ligand 32 **(B)** and standard galantamine **(C)**.

Lead hits from *C. halicacabum* and the standard galantamine were used to estimate the HOMO/LUMO ratio. The DFT-optimized forms and boundary molecular orbitals of the lead-hit ligands (36 and 32) and the standard galantamine using the B3LYP/3–21 G basis set are presented in Figure 11. In drug discovery, Frontier molecular orbitals (HOMO and LUMO) play a crucial role in predicting molecular interactions with biological targets. The HOMO represents the electron-donating ability of the molecule, which influences interactions with nucleophilic residues in the receptor binding site, whereas the LUMO, as the lowest unoccupied molecular orbital, reflects the electron-accepting capability of the molecule, which impacts electrophilic interactions. Table 5 lists key descriptors influencing drug-likeness and biological activity. The HOMO-LUMO energy gap (ΔE) serves as a predictor of molecular stability and reactivity; smaller gaps suggest increased polarizability and potential for stronger biological interactions. Hardness (η) and softness (σ) indicate how easily a molecule adapts to the receptor binding pocket, with softer molecules being more flexible and likely to form stable interactions. The chemical potential (μ) reflects the tendency of electrons to escape from an equilibrium state, influencing the ligand-receptor interaction strength. Electrophilicity (ω) and electronegativity (χ) provide insights into charge transfer, which is critical for stabilizing molecular docking interactions (Choudhary et al., 2019).

**TABLE 5:** Calculated energy gap (ΔE), chemical potential (μ), chemical hardness (η) softness (σ), electronegativity (χ) and electrophilicity (ω) of presence lead hit ligands from *C. halicacabum* and standard galantamine.

Name of the ligand	ΔE (eV)	µ (eV)	η (eV)	σ (eV)	𝒳 (eV)	ω (eV)
Ligand 36	0.02268	−0.19754	0.01134	88.18342	0.5931	0.000221
Ligand 32	0.07135	−0.122205	0.035675	28.030833	0.543265	0.000266
Std galantamine	0.19083	−0.098985	0.095415	10.480532	0.501785	0.000467

Ligand 36, with a high HOMO value, suggests a strong electron donation capacity, potentially enhancing its binding affinity to the receptor. The small energy gap values of ligands 36 (0.02268 eV) and 32 (0.07135 eV) indicate higher molecular reactivity and flexibility, which can improve their adaptability to the active site of the target enzyme. The correlation between the small energy gap and high dipole moment further supports the likelihood of favorable ligand-receptor interactions, which may contribute to enhanced bioactivity of the compounds. By integrating these quantum chemical parameters into the drug discovery process, we can prioritize compounds with optimal electronic properties, thereby improving their potential for strong receptor binding, metabolic stability, and overall pharmacological efficacy. These insights complement our docking and *in vitro* analyses, reinforcing the selection of lead candidates for future development.

## 4 Discussion

The selection of traditional medicinal plants for scientific validation involves a rigorous and systematic approach to identifying and authenticating the therapeutic efficacy of these plants. The process begins with ethnobotanical surveys that document the traditional uses of plants in various cultures. Researchers then prioritize plants based on their historical usage, prevalence, and potential for treating significant health conditions. Once selected, these plants undergo phytochemical screening to identify active compounds that might contribute to their medicinal properties. Promising candidates have advanced to clinical trials, where their efficacy and safety have been evaluated in human subjects (Mukherjee et al., 2022). Throughout this process, collaboration with traditional healers and communities is crucial to ensure respect for indigenous knowledge and sustainable use of plant resources. In this study, the leaves of *C. halicacabum*, commonly known as the balloon vine, showed promise as a source of phytoconstituents that could serve as therapeutic agents for AD. AChE, Aβ, and tau are key proteins implicated in pathophysiology (Majdi et al., 2020). This study aimed to identify natural compounds that could effectively modulate these targets. AChE inhibitors can enhance cholinergic function by preventing the breakdown of acetylcholine, thereby potentially improving cognitive function. Inhibition of amyloid-β peptide aggregation and tau protein stabilization mitigates neurodegenerative processes underlying AD. Although limited, the treatment landscape for AD includes four FDA-approved medications, namely donepezil, tacrine, galantamine, and rivastigmine. Each drug acts as an acetylcholinesterase inhibitor, a class of compound that prevents the breakdown of acetylcholine, a neurotransmitter associated with learning and memory. These medications improve the communication between nerve cells and enhance cognitive performance by increasing acetylcholine levels. Donepezil stands out because it has been specifically approved for the severe stages of AD, making it a vital therapeutic option for patients with advanced symptoms. Although these medications do not cure AD, they can provide symptomatic relief and potentially slow disease progression, thereby improving the patients’ quality of life.

GC-MS provides both qualitative and quantitative information, allowing for precise identification of compounds based on their unique mass spectra. GC-MS analysis identified 40 phytoconstituents, of which 37 were first found in the leaves of *C. halicacabum*. Many of these compounds have not been extensively reported in previous studies, highlighting their potential novelty in neuroprotection. Certain bioactive constituents, such as *Bacopa monnieri*, *Ginkgo biloba*, and *Withania somnifera*, are structurally similar to compounds found in well-known neuroprotective medicinal plants. Several of the newly identified compounds, such as acetic acid, (dodecahydro-7-hydroxy-1,4b,8,8-tetramethyl-10-oxo-2(1H)-phenanthrenylidene)-,2-(dimethylamino)ethyl ester, and 1-(2-hydroxyethoxy)-2-methyldodecane, exhibited stable binding interactions with Aβ), AChE, and tau filaments during molecular docking and molecular dynamics simulations. However, their specific mechanisms of action in AD pathology remain to be explored in further *in vitro* and *in vivo* studies. Thus, this study provides a foundation for future investigations of the neuroprotective potential of *C. halicacabum* and its phytoconstituents.

*In silico* approaches have revolutionized drug discovery by using *in silico* methods streamlining the identification and development of new chemical compounds. These techniques significantly reduce the need for extensive experimental testing by simulating biological interactions and predicting compound efficacy and toxicity, thereby reducing the time and resource costs (Marques et al., 2024). The proposed method is effective in the treatment of various diseases. Notable successes include the development of HIV-1 inhibitors such as atazanavir (Araújo et al., 2021). Overall, *in silico* methods enhance the efficiency of drug development, thereby increasing the likelihood of providing safe and effective medications in the market. *In silico* docking of 40 phytoconstituents identified in the leaves of *C. halicacabum* against three AD receptors, Aβ (PDB ID: 1IYT), AChE (PDB ID: 4EY7), and tau tangle (PDB ID: 5O3L), revealed promising results for the development of potential AD treatments.

Docking analysis provided critical insights into the molecular interactions responsible for ligand stability within the active site. LigPlot analysis of the 1IYT complexes revealed that both ligand 36 and the standard ligand exclusively exhibited hydrophobic interactions, suggesting that non-polar forces play a dominant role in stabilizing these complexes. Ligand 36 formed hydrophobic contacts with Gly33(A), Phe20(A), Val24(A), and Asp23(A), whereas the standard ligand interacted with Asn27(A), Gly29(A), Ile32(A), Ser26(A), Gly25(A), and Lys28(A), indicating potential variations in binding orientation or affinity. The absence of hydrogen bonding in both cases implies that optimizing the hydrogen bond donor/acceptor properties of the ligand could enhance binding stability and specificity. Similarly, in the 4EY7 complex, ligand 36 predominantly formed hydrophobic interactions with Phe297(A), Thr238(A), His405(A), and Val239(A), along with additional contacts involving Leu540(A), Pro537(A), and Trp532(A), emphasizing the role of van der Waals forces in stabilizing the complex, whereas the standard ligand demonstrated both hydrophobic and hydrogen-bonding interactions, including a notable hydrogen bond between Trp182(A) and the ligand at a distance of 2.70 Å, as well as stabilizing hydrophobic interactions with Gln181(A), Phe37(A), Leu178(A), and Gly14(A), suggesting that introducing hydrogen bond donors or acceptors to ligand 36 could enhance its binding stability. Similarly, the interaction analysis of the 5O3L complexes revealed that while both ligand 32 and the standard ligand exhibited hydrogen bonding with Lys331(F), ligand 32 formed a slightly longer bond (3.12 Å) than the standard ligand (2.99 Å), suggesting a marginally weaker hydrogen bond, with ligand 32 engaging multiple hydrophobic residues, including His329(D), Lys340(I), Glu338(I), Val329(G), Val339(E), and Lys340(E), whereas the standard ligand exhibited overlapping hydrophobic interactions with residues such as Lys340(I), Glu338(I), His329(D), and Val339(G), implying comparable binding modes and potential binding affinity. The minor difference in hydrogen bond length could influence ligand stability, underscoring the importance of hydrophobic interactions in ligand binding across different protein complexes, and highlighting the role of hydrogen bonding in modulating stability. This suggests that rational modifications, such as the introduction of hydrogen bond donors or acceptors, could enhance affinity and specificity.

Specifically, the two phytoconstituents exhibited the lowest binding energies against Aβ protein, suggesting a strong inhibitory potential. Additionally, four phytoconstituents showed favorable binding affinities for the AChE protein and six were effective against the tau tangle protein. These findings highlight the potential of phytoconstituents as inhibitors of key AD targets, offering hope to address the hallmark pathology of AD. All top ligands and standard galantamine were further screened for drug likeness, and ADME-tox prediction played a crucial role in assessing the potential efficacy and safety of candidate compounds. Among the top nine ligands evaluated, only six exhibited drug-like properties. ADMET and drug-likeness predictions provide valuable insights into the pharmacokinetic and safety profiles of the studied compounds. However, it is important to acknowledge the potential limitations of these *in silico* predictions as they may not always fully translate into *in vivo* outcomes. Computational models are based on predefined algorithms and training datasets, which may not account for complex biological factors, such as metabolism, transporter-mediated absorption, and tissue-specific distribution. Additionally, inter-individual variability, enzyme polymorphisms, and physiological conditions can significantly influence drug behavior in living systems. Experimental validation through *in vitro* and *in vivo* studies is essential to confirm these predictions and refine the computational models for improved accuracy. A brief discussion of these limitations will strengthen the manuscript by providing a more balanced interpretation of the findings.

Each target lead compound was further screened through MD simulations, and DFT analysis was performed to further explore the stability and electronic properties of the lead hit ligand from each target, i.e., 1IYT-ligand 36. 4EY7-ligand 36 and 5O3L-ligand 32. The RMSD and RMSF values provided crucial insights into the stability and flexibility of the protein-ligand complexes throughout the simulation. RMSD fluctuations reflect the overall stability of the complex, where lower RMSD values typically indicate a more stable interaction. In this study, 4EY7 complexed with ligand 36 exhibited the lowest average RMSD (0.281 nm), suggesting that it formed a highly stable interaction with the protein. In contrast, the 5O3L-ligand 32 complex exhibited a higher RMSD (0.552 nm), indicating greater conformational changes over time. However, its stability was still better than that of the 5O3L-galantamine complex (1.429 nm), suggesting that ligand 32 maintained a reasonable interaction with the protein. The RMSD trend for 1IYT with ligand 36 (0.944 nm) was higher than that of its corresponding standard complex (0.886 nm), indicating slightly greater structural deviations, but still within an acceptable range.

RMSF analysis further elaborated the flexibility of specific residues within the complexes. Lower RMSF values suggested reduced fluctuations and higher stability, whereas higher values indicated greater flexibility. The 1IYT standard complex exhibited a lower RMSF (0.374 nm) than the ligand 36 complex (0.481 nm), implying that the standard ligand contributed to a more stable protein conformation. Similarly, 4EY7 with ligand 36 showed a lower RMSF (0.250 nm) than the standard (0.284 nm), reinforcing the notion that ligand 36 stabilizes the protein-ligand complex. However, the 5O3L-ligand 32 complex exhibited a slightly lower RMSF (0.295 nm) than the standard (0.317 nm), suggesting that ligand 32 stabilizes specific protein regions better than galantamine does.

Fluctuations in RMSD and RMSF values indicated the dynamic behavior of the protein-ligand complexes. Larger deviations may suggest transient conformational changes, which could be attributed to ligand repositioning within the binding site or flexibility in the protein regions critical for ligand interactions. In contrast, complexes with lower and more stable RMSD/RMSF values tended to maintain a consistent binding pose, leading to greater stability. The observed trends highlighted the stabilizing effects of ligand 36 in 4EY7 and its potential as a promising candidate for further investigation. These computational techniques provide deeper insights into the interactions at the molecular level, thereby facilitating the refinement of candidates for further experimental validation.

The HOMO-LUMO gap is a key factor in determining molecular reactivity and interaction potential with biological targets. A smaller energy gap, as observed for ligands 36 and 32, suggests increased polarizability and electronic transitions, which can enhance their adaptability to the active sites of AD-related targets. Additionally, the HOMO represents the molecule’s ability to donate electrons, which may facilitate interactions with nucleophilic residues in the enzyme’s binding pocket. Conversely, the LUMO indicates the electron-accepting capacity of the molecule, which plays a role in binding with electrophilic regions of the target protein. These properties influence the strength and stability of ligand-receptor interactions, potentially improving the inhibition of key enzymes involved in AD pathology. Furthermore, parameters such as hardness (η) and softness (σ) indicate molecular flexibility; softer molecules are more likely to adapt to the receptor-binding pocket, increasing interaction stability. Chemical potential (μ) and electrophilicity (ω) contribute to charge transfer, which is crucial for stabilizing molecular docking interactions. By linking these quantum chemical properties with docking, MD simulations, and *in vitro* results, this manuscript can provide a more comprehensive understanding of how these lead compounds may function as potential AD therapeutics.

*In silico* findings often serve as an essential first step in the research and development process, and provide valuable insights and guidance for subsequent experiments. However, the transition from computational models to real-life applications is complex and requires a thorough experimental validation. This involves testing the hypotheses generated by computer simulations in controlled laboratory settings to assess their efficacy and safety in biological systems. Rigorous experimentation, including *in vitro* and *in vivo* studies, helps to verify computational predictions and ensures that potential treatments and interventions are both effective and safe for human use. The diverse range of bioactive compounds found in plants is a rich resource for the identification of novel therapeutic agents to address the complex pathology of AD. By investing in research focused on natural products, scientists can uncover new possibilities for managing AD, a challenging condition that affects millions of people worldwide. Research in this area may lead to the discovery of novel therapies targeting the underlying mechanisms of AD, such as accumulation of amyloid-β, acetylcholinesterase, and tau protein tangles. These therapies could offer more effective and less invasive treatment options than the current approaches. Moreover, natural products often have fewer side effects, potentially improving patient compliance and overall wellbeing. Ultimately, advancements in this research could contribute to improved patient outcomes, offering hope for those affected by AD, and enhancing their quality of life by preserving cognitive function and independence for longer periods.

## 5 Conclusion

In this study, 40 phytoconstituents were identified in the ethanolic extract of *C. halicacabum* leaves, 37 of which were identified. Further analysis using molecular docking, *in silico* ADMET, and drug-likeness prediction provided insights into their potential therapeutic applications. A comprehensive approach to phytochemistry has identified nine phytoconstituents as potential therapeutic protein targets for AD. Two of these were acetic acid and 2-(dimethylamino)ethyl ester [1R-(1. alpha)], and 1-(2-hydroxyethoxy)-2-methyldodecane, which were screened using MD simulations, LigPlot analysis, and HOMO-LUMO modeling, and compared with standard galantamine. This study has certain limitations, as the high-throughput virtual screening approach based on GC-MS relies on *in silico* predictions, which require further experimental validation. Future research should focus on *in vitro* and *in vivo* studies to confirm the biological activity, pharmacokinetic properties, and safety of these identified compounds. Additionally, structure-activity relationship (SAR) studies and chemical modifications could help optimize their therapeutic potential, paving the way for the development of novel anti-Alzheimer’s drug candidates.

## Data Availability

The raw data supporting the conclusions of this article will be made available by the authors, without undue reservation.

## Abbreviations

AD, Alzheimer’s disease; Aβ, Amyloid-β; AChEIs, Acetylcholinesterase inhibitors; BBD, Box-Behnken design; °C, Celsius; CHARMM-GUI, Chemistry at Harvard Macromolecular Mechanics-Graphical User Interface; Df, Degrees of freedom; DFT, Density Functional Theory; GHz, Gigahertz; GC-MS, Gas Chromatography Mass Spectrometry; iGEMDOCK, Integrated Graphical Environment for Molecular Docking; MD, Molecular Dynamics; MM2, Molecular Mechanics 2 (a force field for molecular modeling); Min, Minutes; Ms, Mean of square; m/z, Mass divided by charge number; NAMD, Nanomolecular Dynamics; ns, Nano second; RSM, Response Surface Methodology; RMSD, Root Mean Square Deviation; SMILES, Simplified Molecular Input Line System; µL, Microlitre; PDB, Protein Data Bank; SS, sum of squares; Tau, Tau protein (microtubule-associated protein).

## References

[B1] Agrawal R. Punarva H. B. Heda G. O. Vishesh Y. M. Karunakar P. (2024). VinaLigGen: a method to generate LigPlots and retrieval of hydrogen and hydrophobic interactions from protein-ligand complexes. J. Biomol. Struct. Dyn. 42 (22), 12040–12043. 10.1080/07391102.2023.2266524 37822199

[B2] Alhazmi H. A. Albratty M. (2022). An update on the novel and approved drugs for Alzheimer disease. Saudi Pharm. J. 30 (12), 1755–1764. 10.1016/j.jsps.2022.10.004 36601504 PMC9805975

[B3] Ameer K. Ameer S. Kim Y. M. Nadeem M. Park M. K. Murtaza M. A. (2022). A hybrid RSM-ANN-GA approach on optimization of ultrasound-assisted extraction conditions for bioactive component-rich Stevia rebaudiana (Bertoni) leaves extract. Foods 11 (6), 883. 10.3390/foods11060883 35327305 PMC8950805

[B4] Andrade S. Nunes D. Dabur M. Ramalho M. J. Pereira M. C. Loureiro J. A. (2023). Therapeutic potential of natural compounds in neurodegenerative diseases: insights from clinical trials. Pharmaceutics 15 (1), 212. 10.3390/pharmaceutics15010212 36678841 PMC9860553

[B5] Araújo J. L. Sousa L. A. Sousa A. O. Bastos R. S. Santos G. T. Lage M. R. (2021). DFT, molecular docking, and ADME/Tox screening investigations of Market-Available drugs against SARS-CoV-2. J. Braz. Chem. Soc. 32 (8), 1628–1641. 10.21577/0103-5053.20210061

[B6] Azad I. (2023). Molecular docking in the study of ligand-protein recognition: an overview. Mol. Docking-Recent Adv. 10.5772/intechopen.106583

[B7] Azargoonjahromi A. (2024). The duality of amyloid-β: its role in normal and Alzheimer’s disease states. Mol. Brain 17 (1), 44. 10.1186/s13041-024-01118-1 39020435 PMC11256416

[B8] Bhadange Y. A. Carpenter J. Saharan V. K. (2024). A comprehensive review on advanced extraction techniques for retrieving bioactive components from natural sources. ACS omega 9 (29), 31274–31297. 10.1021/acsomega.4c02718 39072073 PMC11270575

[B9] Bhagat A. P. Bhuktar A. S. (2020). Phytochemical and pharmacognostic investigation on cardiospermum halicacabum L.(Sapindaceae). J. Pharmacogn. Phytochemistry 9 (3), 1691–1700. 10.1007/978-3-030-14116-5_109-1

[B10] Boateng I. D. (2023). Application of graphical optimization, desirability, and multiple response functions in the extraction of food bioactive compounds. Food Eng. Rev. 15 (2), 309–328. 10.1007/s12393-023-09339-1

[B11] Chaachouay N. Zidane L. (2024). Plant-derived natural products: a source for drug discovery and development. Drugs Drug Candidates 3 (1), 184–207. 10.3390/ddc3010011

[B12] Chen Y. Yu Y. (2023). Tau and neuroinflammation in Alzheimer’s disease: interplay mechanisms and clinical translation. J. neuroinflammation 20 (1), 165. 10.1186/s12974-023-02853-3 37452321 PMC10349496

[B13] Choudhary V. Bhatt A. Dash D. Sharma N. (2019). DFT calculations on molecular structures, HOMO–LUMO study, reactivity descriptors and spectral analyses of newly synthesized diorganotin (IV) 2‐chloridophenylacetohydroxamate complexes. J. Comput. Chem. 40 (27), 2354–2363. 10.1002/jcc.26012 31246313

[B14] Congdon E. E. Ji C. Tetlow A. M. Jiang Y. Sigurdsson E. M. (2023). Tau-targeting therapies for Alzheimer disease: current status and future directions. Nat. Rev. Neurol. 19 (12), 715–736. 10.1038/s41582-023-00883-2 37875627 PMC10965012

[B15] Czech J. Schulz J. B. (2024). Aktuelle medikamentöse Therapieansätze der Alzheimerkrankheit. InFo Neurologie+ Psychiatr. 26 (1), 32–45. 10.1007/s15005-023-3800-z

[B16] Daoud N. E. Borah P. Deb P. K. Venugopala K. N. Hourani W. Alzweiri M. (2021). ADMET profiling in drug discovery and development: perspectives of *in silico*, *in vitro* and integrated approaches. Curr. Drug Metab. 22 (7), 503–522. 10.2174/1389200222666210705122913 34225615

[B17] Divya S. Arivoli S. Samuel T. Raveen R. Jayakumar M. (2018). Gas chromatography-mass sprectometry analysis of cardiospermum halicacabum linnaeus (Sapindaceae) and *Chenopodium album* linnaeus (chenopodiaceae) leaves. J. Pharmacogn. Phytochemistry 7 (3), 2743–2751.

[B18] Dowlath M. J. Karuppannan S. K. Gi D. R. Sb M. K. Subramanian S. Arunachalam K. D. (2020). Effect of solvents on phytochemical composition and antioxidant activity of Cardiospermum halicacabum (L.) extracts. Pharmacogn. J. 12 (6), 1241–1251. 10.5530/pj.2020.12.173

[B19] Durán-Iturbide N. A. Díaz-Eufracio B. I. Medina-Franco J. L. (2020). *In silico* ADME/Tox profiling of natural products: a focus on BIOFACQUIM. ACS omega 5 (26), 16076–16084. 10.1021/acsomega.0c01581 32656429 PMC7346235

[B20] Elangovan A. Ramachandran J. Lakshmanan D. K. Ravichandran G. Thilagar S. (2022). Ethnomedical, phytochemical and pharmacological insights on an Indian medicinal plant: the balloon vine (Cardiospermum halicacabum Linn.). J. Ethnopharmacol. 12 (291), 115143. 10.1016/j.jep.2022.115143 35227784

[B21] Elhawary E. A. Nilofar N. Zengin G. Eldahshan O. A. (2024). Variation of the essential oil components of Citrus aurantium leaves upon using different distillation techniques and evaluation of their antioxidant, antidiabetic, and neuroprotective effect against Alzheimer’s disease. BMC Complementary Med. Ther. 24 (1), 73. 10.1186/s12906-024-04380-x PMC10835836 38308284

[B22] Feng S. Park S. Choi Y. K. Im W. (2023). CHARMM-GUI membrane builder: past, current, and future developments and applications. J. Chem. theory Comput. 19 (8), 2161–2185. 10.1021/acs.jctc.2c01246 37014931 PMC10174225

[B23] Ferrara D. Beccaria M. Cordero C. E. Purcaro G. (2023). Microwave‐assisted extraction in closed vessel in food analysis. J. Sep. Sci. 46 (20), 2300390. 10.1002/jssc.202300390 37654060

[B24] Gee S. Glover K. J. Wittenberg N. J. Im W. (2024). CHARMM‐GUI membrane builder for lipid droplet modeling and simulation. ChemPlusChem 89 (8), e202400013. 10.1002/cplu.202400013 38600039 PMC11324394

[B25] Ghahremanian S. Rashidi M. M. Raeisi K. Toghraie D. (2022). Molecular dynamics simulation approach for discovering potential inhibitors against SARS-CoV-2: a structural review. J. Mol. Liq. 354, 118901. 10.1016/j.molliq.2022.118901 35309259 PMC8916543

[B26] Hasan A. H. Abdulrahman F. A. Obaidullah A. J. Alotaibi H. F. Alanazi M. M. Noamaan M. A. (2023). Discovery of novel coumarin-schiff base hybrids as potential acetylcholinesterase inhibitors: design, synthesis, enzyme inhibition, and computational studies. Pharmaceuticals 16 (7), 971. 10.3390/ph16070971 37513883 PMC10385371

[B27] Jabir N. R. Shakil S. Tabrez S. Khan M. S. Rehman M. T. Ahmed B. A. (2021). *In silico* screening of glycogen synthase kinase-3β targeted ligands against acetylcholinesterase and its probable relevance to Alzheimer’s disease. J. Biomol. Struct. Dyn. 39 (14), 5083–5092. 10.1080/07391102.2020.1784796 32588759

[B28] Jeyadevi R. Sivasudha T. Ilavarasi A. Thajuddin N. (2013). Chemical constituents and antimicrobial activity of Indian green leafy vegetable Cardiospermum halicacabum. Indian J. Microbiol. 53, 208–213. 10.1007/s12088-012-0333-4 24426110 PMC3626954

[B29] Kareti S. R. Subash P. (2020a). *In silico* molecular docking analysis of potential anti-alzheimer's compounds present in chloroform extract of Carissa carandas leaf using gas chromatography MS/MS. Curr. Ther. Res. 1 (93), 100615. 10.1016/j.curtheres.2020.100615 PMC7708940 33306055

[B30] Kareti S. R. Subash P. (2020b). *In silico* exploration of anti-Alzheimer's compounds present in methanolic extract of Neolamarckia cadamba bark using GC–MS/MS. Arabian J. Chem. 13 (7), 6246–6255. 10.1016/j.arabjc.2020.05.035

[B31] Karuppannan S. K. Dowlath M. J. Sb M. K. Gi D. R. Subramanian S. Arunachalam K. D. (2020). Phytochemical and antibacterial activity of Cardiospermum halicacabum against wound pathogens. Pharmacogn. J. 12 (6), 1303–1310. 10.5530/pj.2020.12.179

[B32] Kim S. Oshima H. Zhang H. Kern N. R. Re S. Lee J. (2020). CHARMM-GUI free energy calculator for absolute and relative ligand solvation and binding free energy simulations. J. Chem. theory Comput. 16 (11), 7207–7218. 10.1021/acs.jctc.0c00884 33112150 PMC7658063

[B33] Mahnashi M. H. Alyami B. A. Alqahtani Y. S. Alqarni A. O. Jan M. S. Ayaz M. (2021). Neuroprotective potentials of selected natural edible oils using enzyme inhibitory, kinetic and simulation approaches. BMC Complementary Med. Ther. 21, 248–254. 10.1186/s12906-021-03420-0 PMC8487577 34600509

[B34] Majdi A. Sadigh-Eteghad S. Rahigh Aghsan S. Farajdokht F. Vatandoust S. M. Namvaran A. (2020). Amyloid-β, tau, and the cholinergic system in Alzheimer’s disease: seeking direction in a tangle of clues. Rev. Neurosci. 31 (4), 391–413. 10.1515/revneuro-2019-0089 32017704

[B35] Marques L. Costa B. Pereira M. Silva A. Santos J. Saldanha L. (2024). Advancing precision medicine: a review of innovative *in silico* approaches for drug development, clinical pharmacology and personalized healthcare. Pharmaceutics 16 (3), 332. 10.3390/pharmaceutics16030332 38543226 PMC10975777

[B36] Mathew A. Balaji E. V. Pai S. R. Kishore A. Pai V. Pemmireddy R. (2023). Current drug targets in Alzheimer’s associated memory impairment: a comprehensive review. CNS and Neurological Disorders-Drug Targets-CNS and Neurological Disord. 22 (2), 255–275. 10.2174/1871527321666220401124719 35366787

[B37] Menichini F. Losi L. Bonesi M. Pugliese A. Loizzo M. R. Tundis R. (2014). Chemical profiling and *in vitro* biological effects of Cardiospermum halicacabum L.(Sapindaceae) aerial parts and seeds for applications in neurodegenerative disorders. J. enzyme inhibition Med. Chem. 29 (5), 677–685. 10.3109/14756366.2013.840614 24102527

[B38] Mukherjee P. K. Banerjee S. Gupta B. D. Kar A. (2022). Evidence-based validation of herbal medicine: translational approach. InEvidence-based validation Herb. Med. 1, 1–41. 10.1016/b978-0-323-85542-6.00025-1

[B39] Mumit M. A. Pal T. K. Alam M. A. Islam M. A. Paul S. Sheikh M. C. (2020). DFT studies on vibrational and electronic spectra, HOMO–LUMO, MEP, HOMA, NBO and molecular docking analysis of benzyl-3-N-(2, 4, 5-trimethoxyphenylmethylene) hydrazinecarbodithioate. J. Mol. Struct. 15, 1220–128715. 10.1016/j.molstruc.2020.128715 PMC7305508 32834109

[B40] Mustapha M. Taib C. N. (2022). Beneficial role of Vitexin in Parkinson’s disease. Malays. J. Med. Sci. MJMS 30 (2), 8. 10.21315/mjms2023.30.2.2 37102042 PMC10125247

[B41] Ornish D. Madison C. Kivipelto M. Kemp C. McCulloch C. E. Galasko D. (2024). Effects of intensive lifestyle changes on the progression of mild cognitive impairment or early dementia due to Alzheimer’s disease: a randomized, controlled clinical trial. Alzheimer's Res. and Ther. 16 (1), 122. 10.1186/s13195-024-01482-z 38849944 PMC11157928

[B42] Pardo-Moreno T. González-Acedo A. Rivas-Domínguez A. García-Morales V. García-Cozar F. J. Ramos-Rodríguez J. J. (2022). Therapeutic approach to Alzheimer’s disease: current treatments and new perspectives. Pharmaceutics 14 (6), 1117. 10.3390/pharmaceutics14061117 35745693 PMC9228613

[B43] Pedroza P. Miller-Petrie M. K. Chen C. Chakrabarti S. Chapin A. Hay S. (2022). Global and regional spending on dementia care from 2000–2019 and expected future health spending scenarios from 2020–2050: an economic modelling exercise. EClinicalMedicine 45, 101337. 10.1016/j.eclinm.2022.101337 35299657 PMC8921543

[B44] Pinlap B. R. Kamdem B. P. Kamto E. L. Ngouana V. Dongmo Y. K. Lunga P. K. (2024). Extracts from Cardiospermum grandiflorum and Blighia welwitschii (Sapindaceae) reveal antibacterial activity against Shigella species. South Afr. J. Bot. 164 (1), 419–428. 10.1016/j.sajb.2023.12.010

[B45] Razzaghi M. Sheibani F. Kimia N. Razzaghi Z. Chenari Z. Ashrafi F. (2024). Photobiomodulation's potential as a non-invasive therapy for alzheimer's disease and minimal cognitive impairment: a 12-week investigation. Photodiagnosis Photodyn. Ther. 46, 103991. 10.1016/j.pdpdt.2024.103991 38367922

[B46] Reddy H. Kshirsagar S. Islam M. A. Baig J. Pradeepkiran J. A. (2025). Amyloid-β and phosphorylated tau are the key biomarkers and predictors of Alzheimer’s disease. Aging Dis. 16 (2), 658–682. 10.14336/AD.2024.0286 PMC11964437 38739937

[B47] Saikia A. Chetia A. Sultana H. Khan N. J. (2023). A review on phytochemical and pharmacological activities of cardiospermum halicacabum linn. 12(11):1–10.

[B48] Saini R. S. Binduhayyim R. I. Gurumurthy V. Alshadidi A. A. Bavabeedu S. S. Vyas R. (2024). *In silico* assessment of biocompatibility and toxicity: molecular docking and dynamics simulation of PMMA-based dental materials for interim prosthetic restorations. J. Mater. Sci. Mater. Med. 35 (1), 28. 10.1007/s10856-024-06799-7 38833196 PMC11150300

[B49] Salvoza N. Giraudi P. J. Tiribelli C. Rosso N. (2022). Natural compounds for counteracting nonalcoholic fatty liver disease (NAFLD): advantages and limitations of the suggested candidates. Int. J. Mol. Sci. 23 (5), 2764. 10.3390/ijms23052764 35269912 PMC8911502

[B50] Sarkar S. Bhui U. Kumar B. Ashique S. Kumar P. Sharma H. (2024). Correlation between cognitive impairment and peripheral biomarkers-significance of phosphorylated tau and amyloid-β in Alzheimer’s disease: a new insight. Curr. Psychiatry Res. Rev. 21. 10.2174/0126660822329981241007105405

[B51] Segura L. Santos N. Flores R. Sikazwe D. McGibbon M. Blay V. (2024). Exploring tau fibril-disaggregating and antioxidating molecules binding to membrane-bound amyloid oligomers using machine learning-enhanced docking and molecular dynamics. Molecules 29 (12), 2818. 10.3390/molecules29122818 38930883 PMC11206291

[B52] Shabangu K. P. Chetty M. Bakare B. F. (2024). Optimization and modeling of a dual-chamber microbial fuel cell (dcmfc) for industrial wastewater treatment: a box–behnken design approach. Energies 17 (11), 2740. 10.3390/en17112740

[B53] Shukla D. Suryavanshi A. Bharti S. K. Asati V. Mahapatra D. K. (2024). Recent advances in the treatment and management of alzheimer's disease: a precision medicine perspective. Curr. Top. Med. Chem. 24 (19), 1699–1737. 10.2174/0115680266299847240328045737 38566385

[B54] Sonntag N. O. (1982). Glycerolysis of fats and methyl esters—status, review and critique. J. Am. Oil Chemists' Soc. 59 (10), 795A–802A. 10.1007/bf02634442

[B55] Subash P. Rao K. S. (2021). *In silico* molecular docking analysis for potential anti-Alzheimer's compounds from the methanolic leaf extract of Erythroxylum monogynum using Gas chromatography–mass spectrometry. J. Saudi Chem. Soc. 25 (8), 101285. 10.1016/j.jscs.2021.101285

[B56] Sun D. Gao W. Hu H. Zhou S. (2022). Why 90% of clinical drug development fails and how to improve it? Acta Pharm. Sin. B 12 (7), 3049–3062. 10.1016/j.apsb.2022.02.002 35865092 PMC9293739

[B57] Sun T. Zhen T. Harakandi C. H. Wang L. Guo H. Chen Y. (2024). New insights into butyrylcholinesterase: pharmaceutical applications, selective inhibitors and multitarget-directed ligands. Eur. J. Med. Chem. 275, 116569. 10.1016/j.ejmech.2024.116569 38852337

[B58] Terao I. Kodama W. (2024). Comparative efficacy, tolerability and acceptability of donanemab, lecanemab, aducanumab and lithium on cognitive function in mild cognitive impairment and Alzheimer's disease: a systematic review and network meta-analysis. Ageing Res. Rev. 20, 102203. 10.1016/j.arr.2024.102203 38253184

[B59] Thandivel S. Rajan P. Gunasekar T. Arjunan A. Khute S. Kareti S. R. (2024). *In silico* molecular docking and dynamic simulation of anti-cholinesterase compounds from the extract of Catunaregam spinosa for possible treatment of Alzheimer's disease. Heliyon 10 (7), e27880. 10.1016/j.heliyon.2024.e27880 38560123 PMC10981039

[B60] Topiya H. R. Pandya D. J. (2024). Karnasphota: a neuroprotective herb from the treasure of Ayurveda. Curr. Tradit. Med. 10 (7), 46–52. 10.2174/2215083810666230905152757

[B61] Vanaja P. Moorthy N. S. Rajpoot V. S. Rao H. Goswami R. K. Subash P. (2025). Metabolite profiling, antimalarial potentials of Schleichera oleosa using LC-MS and GC-MS: *in vitro*, molecular docking and molecular dynamics. Front. Mol. Biosci. 12, 1543939. 10.3389/fmolb.2025.1543939 40026697 PMC11867967

[B62] Vázquez J. López M. Gibert E. Herrero E. Luque F. J. (2020). Merging ligand-based and structure-based methods in drug discovery: an overview of combined virtual screening approaches. Molecules 25 (20), 4723. 10.3390/molecules25204723 33076254 PMC7587536

[B63] Zang E. Qiu B. Chen N. Li C. Liu Q. Zhang M. (2021). Xanthoceras sorbifolium Bunge: a review on botany, phytochemistry, pharmacology, and applications. Front. Pharmacol. 12, 708549. 10.3389/fphar.2021.708549 34526898 PMC8435727

[B64] Zou J. P. Zhang X. Q. Guo Q. W. Xu X. H. Liu W. W. Zhang F. (2024). Synthesis, biological activity, X-ray crystallographic, DFT calculations and molecular dynamics simulation studies of 2-phenylthiazole-1, 3, 5-triazine derivatives as potential cholinesterase inhibitors. J. Mol. Struct. 5, 1309–138203. 10.1016/j.molstruc.2024.138203

