# Supplementary material for: Virtual screening and molecular dynamics of anti-Alzheimer compounds from Cardiospermum halicacabum via GC-MS
Source: Front Chem. 2025 Apr 4;13:1586728. doi: 10.3389/fchem.2025.1586728 (PMC12006154; doi:10.3389/fchem.2025.1586728)

**SUPPLEMENTARY FILE**

**Virtual screening and molecular dynamics of anti-Alzheimer's compounds from *Cardiospermum halicacabum* via GC-MS.**

Selvan Kaviyarasu^1^; Nallamuthu Padmanaban^1^; Sulekha Khute^1^, Gokhan Zengin^2^, Paranthaman Subash^1*^.

1.Department of Pharmacognosy, Sri Shanmugha College of Pharmacy, Sankari, Salem

district – 637 304, Tamil Nadu, India

2. Department of Biology, Science Faculty, Selcuk University, Campus, Konya, Turkey

Corresponding author

Dr. Paranthaman Subash,

Professor and Head,

Department of Pharmacognosy,

Sri Shanmugha College of Pharmacy,

Sankari, Salem district, 637 304, Tamil Nadu, India

Email: [subashpharm@gmail.com](mailto:subashpharm@gmail.com) and [subash.pharmacy@shanmugha.edu.in](mailto:subash.pharmacy@shanmugha.edu.in)

Phone: +91-9442960131.

Orcid ID: 0000-0001-8060-1591

**Fig S1**: Authentication certificate *C. halicacabum*

**Supplementary Table S1:** Independent and dependent variable of box behnken design for optimization of sample

| **A definitive coding value is used** |
| --- |
| **Factors Low (-1) Medium (0) High (+1)** |
| **Independent factors**  X_1_= Weight of powder (gm) 0.5 1 1.5  X_2_= Solvent Volume (ml) 5 10 15  X_3_= Extraction time (min) 5 10 15 |
| **Dependent factors**  Y_1_= % Yield (gm) 0.009 0.34 0.4 |

**Supplementary Table S2:** Summary result of regression analysis for variables like Df, SS, Ms, F, *þ, R ^2^* SD.

| **Variables** | **Df** | **SS** | **Ms** | **F** | ***þ*** | **R^2^** | **SD** | | **Goodness of fit** |
| --- | --- | --- | --- | --- | --- | --- | --- | --- | --- |
| **% Yield (gm)** | | | | | | | | | |
| **Model** | 9 | 0.1408 | 0.0156 | 5.59 | < 0.0168 | 0.8778 | | 0.0529 | Significant |
| **Residual** | 7 | 0.0196 | 0.0028 | - | - | - | | - |  |
| **Total** | 16 | 0.1604 | - | - | - | - | | - |  |

Degrees of freedom (Df), Sum of squares (Ss), Mean of squares (Ms), p-value, F-value, R2, and standard deviation (SD).

**Supplementary Table S3:** Reported Phytoconstituents from the *C. halicacabum* leaves

| **S.NO** | **Reported Phytoconstituents** |
| --- | --- |
| **(Dowlath et.al., 2020)** | |
|  | Cyclopentanamine |
|  | Glycerin |
|  | Hexadecanoic acid, ethyl ester |
|  | **Neophytadiene** |
|  | Squalene |
|  | Hexacosane |
|  | 2,3-Dihydro-benzofuran |
|  | **3,7,11,15-Tetramethyl-2-hexadecen-1-ol** |
|  | 1,4,5,6-Tetrahydropyrimidine |
|  | Pentadecanoic acid, 14-methyl-, methyl ester |
|  | 9-Octadecenoic acid, methyl ester |
|  | 1,2-Dihydro-8-hydroxylinalool |
|  | Octadecanoic acid, ethyl ester |
|  | 9,12-Octadecadienoic acid |
|  | 9,12,15-Octadecatrienoic acid, methyl ester |
|  | Cyclopropanebutanoic acid |
|  | 11,14-Eicosadienoic acid, methyl ester |
|  | 9-Octadecenoic acid |
|  | 1,2-Benzenedicarboxylic acid, dibutyl ester |
|  | 14-. Beta. -H-pregna |
|  | 4,8,12,16-Tetramethylheptadecan-4-olide |
|  | (9Z,12Z,15Z)-2-Hydroxy-3-Methoxypropyl Octadeca-9,12,15-Trienoate |
|  | 2-[(triethylsilyl)oxy] ethanol |
|  | 2-tridecenal |
|  | Tetratetracontane |
|  | 9-octadecenamide |
|  | 2-methyloctacosane |
|  | 2,4-ditert-butylphenol |
|  | E-14-hexadecenal |
|  | 1-nonadecene |
|  | 1-hexadecene |
|  | Pentacosane |
|  | Octacosane |
|  | E-15-heptadecenal |
|  | Tritetracontane |
|  | Tetradecanoic acid, 12-methyl-, methyl ester |
|  | Octadecanamide |
|  | 1-tetradecene |
|  | 9,12,15-octadecatrienoic acid |
|  | N-hexadecanoic acid |
|  | Heneicosane |
|  | Eicosane |
|  | 2-hexadecen-1-ol, 3,7,11,15-tetramethyl |
|  | 2-pentadecanone, 6,10,14-trimethyl |
|  | Bicyclo[7.2.0]undec-4-ene, 4,11,11-trimethyl-8-methylene |
|  | Tetradecanamide |
|  | Hexadecanoic acid, methyl ester |
| **(Jeyadevi et.al., 2013)** | |
|  | Cyclohexane-1,4,5-triol-3-one1-carboxylic acid |
|  | 1-hydroxytetradecane |
|  | 11-trimethyl-8-methylene |
|  | Caryophyllene |
|  | N-methyl tomatidine |
|  | 3-methylbutanamide |
|  | Phenylethyl alcohol |
|  | Alpha-octadecene |
|  | Hexadecene |
|  | Nonadecene |
|  | Heptadecane |
|  | 1,2-benzenedi carboxylic acid |
|  | Benzene acetic acid |
|  | 14-methyl-8-hexadecyne |
|  | Benzaldehyde |
|  | 1-tert-butyl-2-methoxy-4-methyl-3, 5-dinitrobenzene |
|  | **Phytol** |
|  | Beta-phenethylphenyl acetate |
|  | 2-phenylethyl ester |
|  | Hexadeconoic acid |
|  | Octadeconoic acid |
|  | 2-nitro-2-(30-hydroxybutyl) Cyclododecanone |
| **(Subramanyam et al., 2007)** | |
|  | Β-Arachidic acid |
|  | Apigenin |
|  | Apigenin-7-O-glucuronide |
|  | Chrysoeriol-7-O-glucuronide |
|  | Luteolin-7-Oglucuronide |
|  | Saponin |
|  | L-amino acid |
|  | Β sitosterol |
|  | Quebrachitol |
|  | Capric acid |
|  | Arachidic fatty acid |
|  | Dl-dopa |
|  | 1 6 10-dodecatrien 7 11-dimethyl-3-methylene- (e)- |
|  | Phenol, 2,6-bis(1,1-dimethylethyl)-4-methyl-, methylcarbamate |
|  | 1,14-tetradecanediol |
|  | Pseudoephedrine |
|  | 2-propenamide |
|  | N-[2-(dimethylamino)ethyl] |
|  | E-2-octadecadecen-1-ol |
|  | **(Saikia et.al., 2023)** |
|  | 1,2,4-Trioxolane-2-octanoic acid, 5-octyl-, methyl ester |
|  | Ricinolenic acid |
|  | [1,1-bicyclopopyl] |
|  | 2-[9-octadecenyloxyl] |
|  | 2-hexyl-methyl ester |
|  | 11-octadecenoic acid |
|  | Methyl ester |
|  | 7-methyl-7-tetradecan1-ol acetate |
|  | Oleic acid |
|  | 1,2,3-propanetriyl ester |
| **(Karuppannan et.al., 2020)** | |
|  | 2(5H)-furanone, 5-methyl |
|  | 3-Pyrrolidinopropionitrile |
|  | 1,5-Anhydro-6-deoxyhexo-2,3-  Diulose |
|  | (4-Aminophenyl)(2-methylpiperidin-1-yl) Methanone |
|  | Undecanoic acid |
|  | 2(4h)-Benzofuranone,  5,6,7,7a-tetrahydro6-hydroxy-4,4,7atrimethyl-, (6s-cis)- |
|  | 9,12-Octadecadienoic acid, methyl ester |
|  | Hexadecanoic acid, 2-hydroxy-1-  (hydroxymethyl) ethyl ester |
|  | 9,12-Octadecadienoic acid (Z,Z)-, 2-hydroxy-  11-(hydroxymethyl)ethyl ester |
|  | Methyl (Z)-5,11,14,17-eicosatetraenoate |
|  | Vitamin E |
|  | **(Bhagat and Bhuktar, 2020)** |
|  | 3-O-Methyl-d-glucose |
|  | Mannofuranoside, 1-O-decyl |
|  | 2(3H)-Naphthalenona, 3[{(1,1-dimethylethyl)dime thylsilyl]4,4a,5,6,7,8-hexah ydro-1,4a-dimethyl-7 |
|  | 18-hydroxy-10-pentyl-11-o xa-1,5-ditha-spirononadec-one |
|  | Beta-Sitosterol |
|  | R-Sitosterol |
|  | 9-octadecenoic acid, 2-hydroxy-1-(hydroxymethyl)ethyl ester |
|  | Phthalic acid, isobutyl octadecyl ester |
|  | Phthalic acid, butyl tetradecyl ester |
|  | Stigmasterol |
|  | 9,19-Cyclolanostane-3,7-diol |
| **(Divya et.al.,2018)** | |
|  | Methylene chloride |
|  | 11-Hexadecen-1-ol, (Z)- |
|  | 1-Hexyl-2-nitrocyclohexane |
|  | 2(3h)-furanone |
|  | D-mannitol |
|  | Hexadecanal |
|  | Naproxen |
|  | Β-D-Glucopyranoside, methyl |
|  | Methyl decanoate |
|  | 11-Eicosenoic acid, (Z)- |
|  | 7-Oxodehydroabietic acid, trimethylsilyl ester |
|  | β Carotene |
|  | Coumarin |
| **Currently identified Phytoconstituents from *C. halicacabum* by GCMS** | |
|  | 1,1-Diethoxy-2-Butene |
|  | 2-Methyl-1,3-Dioxolan-4-One |
|  | Hydroperoxide, 1-Methylethyl |
|  | Pentane, 1,5-Dibromo |
|  | Cyclododecanone |
|  | Cyclopropane |
|  | 3-Hexanone, 2,2-Dimethyl- |
|  | Methacryloyl Chloride |
|  | (2e)-2-Butenoyl Chloride |
|  | Vinyl (2e)-2-Butenoate |
|  | 2-Nitro-1-Octanol |
|  | 4-Allyl-2-T-Butyl-3-(Naphthalene-2-Carbonyl)Oxazolidin-5-One |
|  | 3-Hexanone, 2,2-Dimethyl- |
|  | (-)-5-Oxatricyclo[8.2.0.0(4,6)]Dodecane,,12-Trimethyl-9-Methylene-, [1r-(1r*,4r*,6r*,10s*)]- |
|  | Methyl Hexofuranoside |
|  | 1,3-Dioxane, 2-(1,3-Dioxolan-2-Yl)- |
|  | (Z)-6-(Tetrahydropyran-2-Yloxy)-1-Trimethylsilylhex-2-Ene |
|  | Benzene, Pentachloro(Trichloroethenyl) |
|  | Propane, 2-Fluoro-2-Methyl |
|  | Propane, 2-Methoxy-2-Methyl |
|  | Tetradecane, 2-Methyl |
|  | (1e)-1-Nitro-1-Propene |
|  | **Neophytadiene** |
|  | 2-Undecene, 9-Methyl-, (Z) |
|  | **3,7,11,15-Tetramethyl-2-Hexadecen-1-Ol** |
|  | D-Norleucine |
|  | Butanoic Acid |
|  | Oxalic Acid, Dineopentyl Ester |
|  | Methacrylic Anhydride |
|  | **Phytol** |
|  | Bis(2-(Dimethylamino)Ethyl) Ether |
|  | 1-(2-Hydroxyethoxy)-2-Methyldodecane |
|  | 8-Methyl-7-Nonen-5-Olide |
|  | 3-Methoxysulpholane |
|  | 3-Cyclopentylpropionic Acid, 2-Dimethylaminoethyl Ester |
|  | Acetic Acid, (Dodecahydro-7-Hydroxy-1,4b,8,8-Tetramethyl-10-Oxo-2(1h)-Phenanthrenylidene)-, 2-(Dimethylamino)Ethyl Ester, [1R-(1.Alpha)] |
|  | Methoxyacetic Acid, 2-Tetrahydrofurylmethyl Ester |
|  | 1,2-Benzenedicarboxylic Acid, Diisooctyl Ester |
|  | N-(2,6-Dimethyl-Phenyl)-N-(2-Morpholin-4-Yl-2-Phenyl-Acetyl)-Benzamide |
|  | 2(3H)-Furanone, Dihydro-3-Hydroxy-4,4-Dimethyl |


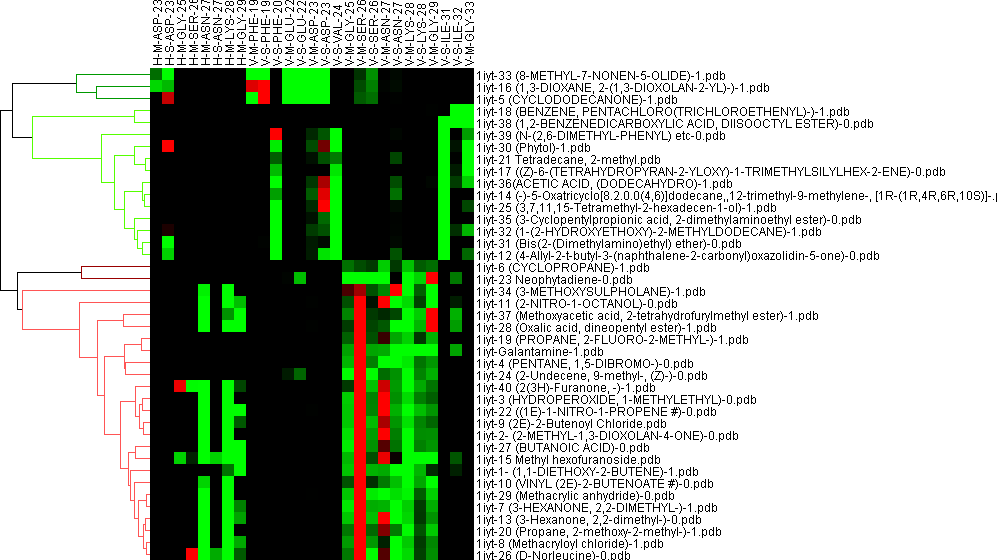


**Fig S2**: The hierarchical clustering results from *C. halicacabum* phytotoconstitunets against 1IYT (a); 4EY7 (b) and 5O3L (c)


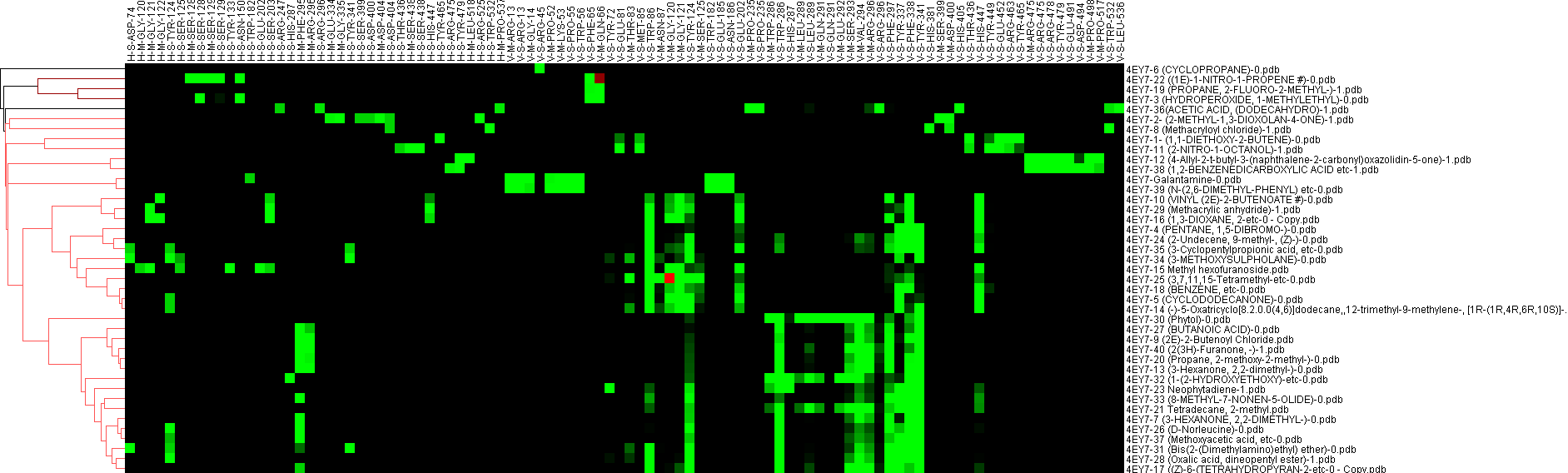


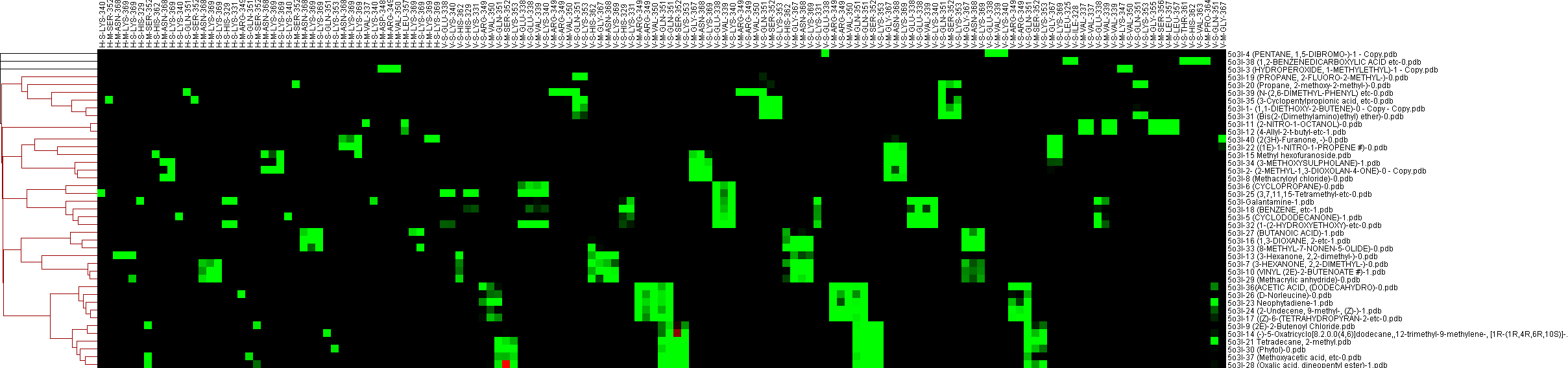

Supplement: Supplementary file 1 [file DataSheet1.docx]
